# How the Piecewise-Linearity Requirement for the Density Affects Quantities in the Kohn–Sham System

**DOI:** 10.1021/acs.jctc.4c01152

**Published:** 2024-12-16

**Authors:** Eli Kraisler

**Affiliations:** Fritz Haber Center for Molecular Dynamics and Institute of Chemistry, The Hebrew University of Jerusalem, 9190401 Jerusalem, Israel

## Abstract

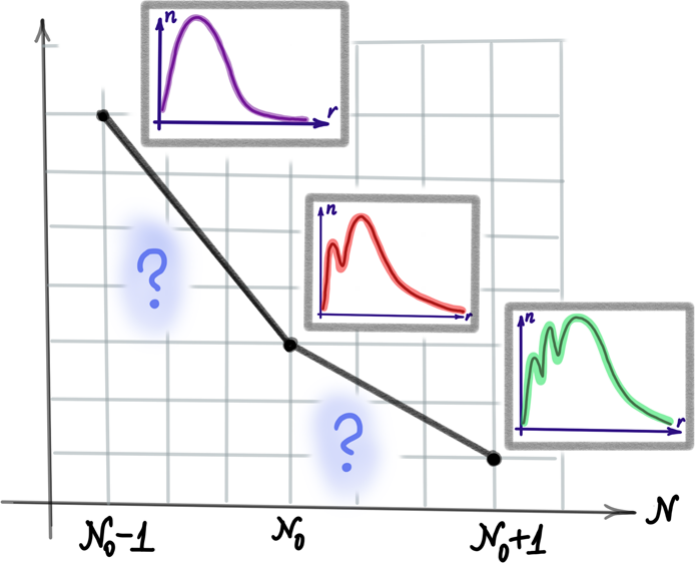

Kohn–Sham (KS) density functional theory (DFT) is an extremely popular, in-principle exact method, which can describe any many-electron system by introducing an auxiliary system of noninteracting electrons with the same density. When the number of electrons, *N*, changes continuously, taking on both integer and fractional values, the density has to be piecewise-linear, with respect to *N*. In this article, I explore how the piecewise-linearity property of the exact interacting density is reflected in the KS system. In particular, I suggest to express KS quantities using the two-point Taylor expansion in *N* and find how the expansion coefficients are restricted by the piecewise-linearity requirement. Focus is given to the total electron density, the KS subdensities, and the highest occupied (HOMO) orbital density. In addition to exact analytical results, common approximations for the HOMO, namely, the frozen and the linear regimes, are analyzed. A numerical investigation using various exchange-correlation approximations is performed to test the analytical findings. The outcomes of this work will help to remove density-driven errors in DFT calculations for open systems and ensembles.

## 1 Introduction

Kohn–Sham density functional theory (KS-DFT) is a leading approach in theoretical chemistry, solid state physics and materials science.^1−4^ It has been applied to a variety of many-electron systems, from atoms and small molecules to macro-molecules, nanoparticles and solids. Being exact in principle and approximate in practice, the success of a DFT-based calculation crucially depends on the accuracy of the exchange-correlation (xc) approximation employed. In the last six decades, hundreds of xc functionals have been suggested and tested for different systems, showing varying degrees of accuracy and transferability.

One way to create predictive xc approximations from first principles is by satisfying exact constraints.^5−7^ The idea relies on the fact that while the exact xc functional is not known, certain formal properties of many-electron systems are. Developing an xc approximation that incorporates known properties by construction is expected to yield high accuracy together with a wide range of applicability.

One exact property of many-electron systems is piecewise linearity in ensemble states. The seminal work of Perdew, Parr, Levy and Balduz^8^ shows that for systems with a varying, possibly fractional number of electrons, *N* = *N*_0_ + α (where *N*_0_ ∈  and α ∈ [0, 1]), the ground state at zero temperature is described by the two-state ensemble,^9^ Λ̂, where
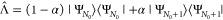
1Here |Ψ_*N*_0_+*p*_⟩ is the ground state of a system with *N*_0_ + *p* electrons (here and below, *p* = 0 or 1),^10^ and the statistical weights of this ensemble, (1 – α) and α, satisfy the following properties: (a) they sum up to 1; (b) they yield the correct number of electrons, namely Tr{Λ̂ *N̂*} = (1 – α) *N*_0_ + α (*N*_0_ + 1) = *N*_0_ + α. For example, the ground state of the Li atom with 2.6 electrons is an ensemble of the ground state of Li^+^ and that of Li, with weights of 0.4 and 0.6, respectively.

As a direct result of eq 1, the total energy of the system, the ensemble electron density and any property that is described by an operator, is piecewise linear in α, i.e., in *N*. The above statements are exact for any *N*-electron system. Therefore, they must be reproduced by a DFT solution with the exact xc functional; approximate functionals should try to mimic this behavior.

Well-known is piecewise-linearity of the total energy: Between any two adjacent integer values of *N*, the energy is linear, with the slope being the negative of the appropriate ionization potential (IP). As *N* crosses an integer, the slope value is discontinuous; the change in the slope equals the fundamental gap. Enforcing piecewise-linearity in an approximate functional–e.g., by tuning a parameter,^11−14^ by an ensemble generalization^15^ or by developing a new functional^16−18^ – can have an enormous, positive effect (see, e.g.,^11,12,19−32^ and references therein). Fully or even partially enforced piecewise-linearity of the energy can significantly improve the prediction of the IP via the highest occupied KS eigenvalue, and the fundamental gap of finite systems, it can introduce the missing derivative discontinuity^8,33−35^ and create the desirable spatial jump in the KS potential. Furthermore, it can prevent delocalization and resolve the spurious fractional dissociation problem.

In contrast, until recently,^36,37^ piecewise-linearity of the electron density received much less attention. In a recent paper^37^ (Section VII), it has been shown that by enforcing piecewise-linearity of the density in an atomic system with a fractional *N*, and obtaining the corresponding KS potential by numerical inversion, one can introduce a plateau to the KS potential and improve the potential’s asymptotic decay (termed there “inverted xc”). This is achieved even when the underlying xc approximation, which was used to obtain electron densities at integer *N*, is as simple as the local density approximation (LDA).^38^ However, deeply understanding and fully utilizing the piecewise-linear properties of the density in the context of KS-DFT is a goal yet to be achieved. In this contribution, a step toward this goal is made, inspired by the advice expressed in ref (39), *to closely look at the density*.

The central question of this paper is: how the requirement of piecewise-linearity of the density for a finite system with fractional *N* is reflected in the corresponding KS system? In particular, since the KS potential, , is α-dependent, so are the KS eigenvalues, orbitals and other resultant quantities. But if the density constructed within the KS system must be strictly linear in α, how does this restrict the α-dependence of other KS quantities?

I address the aforementioned question by expanding KS quantities in terms of α, using the two-point Taylor expansion (2pTE) method in Section 2. By doing so and requiring piecewise-linearity, I obtain relations between the expansion coefficients of the auxiliary subdensities  and  (defined in eq 5 below). Surprisingly, their difference, which is by definition the orbital density of the highest occupied level (HOMO), remains unconstrained. Therefore, common approximate regimes for the HOMO, namely the frozen and the linear regimes, are considered. Furthermore, in Section 3 the question is explored numerically, using common xc functionals in atomic systems. Surprisingly, whereas for some atoms the achieved analytical results are accurately reproduced, for atoms in Group 1 of the Periodic Table the first derivative of the HOMO orbital density diverges as the electron number approaches an integer from above. Section 4 is devoted to discussion of the obtained results, trying to deeper understand the difference between the typical cases (represented in Section 3.2 by the Ag atom) and the atypical results (represented in Section 3.3 by Li). A summary and outlook are provided in Section 5.

## 2 Theory

### 2.1 Essential Background

A finite, interacting many-electron system with a fractional electron number, *N*, is described at zero temperature by the ensemble of eq 1. As a result, its density equals
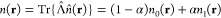
2where *n̂*(**r**) is the density operator and *n*_*p*_(**r**) = ⟨Ψ_*N*_0_+*p*_|*n̂*(**r**)|Ψ_*N*_0_+*p*_⟩ is the ground-state density of the system with (*N*_0_ + *p*) electrons (recall that *p* = 0 or 1). Obviously, *n*_*p*_(**r**) do not depend on α.

To describe a fractional-*N* system with KS-DFT, one can pursue one of the following two approaches. The first approach is to separately address the *N*_0_- and (*N*_0_ + 1)-systems, get the density for each, and then obtain the ensemble density using eq 2. The result will be piecewise-linear, by construction, and any deviation from the exact ensemble density would stem from inaccuracies in *n*_*p*_(**r**). This approach involves two independent calculations, i.e., two KS systems. Within this approach, one can also numerically obtain the KS potential that corresponds to the aforementioned ensemble density, using one of the numerical inversion algorithms.^40−42^ Subsequently, the KS orbitals, eigenvalues and other derived properties can be calculated. Recently, this allowed to numerically study^37^ the spatial steps that emerge in the KS potential as a direct result of the piecewise linearity of the density, even if the underlying approximation is as simple as the LDA. Following the terminology of ref (37), we term such potentials here “inverted xc” potentials. Particularly, we employ “inverted LDA” (invLDA) and “inverted PBE” (invPBE).

The second approach, which we describe below, uses one KS system. Relying on refs.,^43−47^ we know that *n*(**r**) of eq 2 is noninteracting ensemble *v*-representable. Therefore, there exists a KS potential, , whose ensemble ground state yields *n*(**r**). It is emphasized that *v*_KS_ depends on α: for α = 0, it reconstructs *n*_0_(**r**), for α = 1 it reconstructs *n*_1_(**r**), and for fractional values of α – their linear combination, as in eq 2. Generally, there is no reason to assume that the α-dependence of *v*_KS_ is negligible: on the contrary, for low values of α, *v*_KS_ experiences abrupt spatial changes, namely a plateau that tends to infinity for α → 0^+^.^37,48−52^ As a result, the KS eigenvalues, , orbitals  and all derived quantities depend on α, which is emphasized by the superscript (α).

As we come to describe *n*(**r**) within the KS system, we immediately realize that the KS system *must* be in an ensemble state: since the number of electrons in the KS system is fractional, the KS ground state cannot be a pure state. The KS ensemble therefore equals
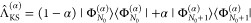
3with |Φ_*N*_0_+*p*_^(α)^⟩ being the ground state of the KS potential , with *N*_0_ + *p* electrons. Therefore,  and  are Slater determinants constructed of the same KS orbitals , which all correspond to the same KS potential, ;  differs from  by having one more row and one more column.

The KS ensemble density that follows from eq 3 is
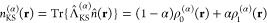
4where
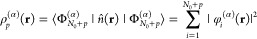
5Performing another algebraic step, one can also express the density with fractional occupations, *f*_*i*_:  with the first *N*_0_ levels being fully occupied, *f*_*N*_0_+1_ = α, and all the higher-lying levels being vacant. It should be clearly emphasized that all the above steps, including the introduction of fractional occupations, are not a matter of choice: they all inevitably follow from the decision of describing a system with fractional *N* using one KS system.

The auxiliary subdensities  and  are the sums of the first *N*_0_ and *N*_0_ + 1 squared KS orbitals, respectively. These are internal quantities of the KS system; generally, they are not directly related to any physical quantity. Only at the edges of the α-range,  and . Nonetheless, ρ_0_ and ρ_1_ are of particular interest, as these and related quantities frequently appear as ingredients in xc approximations (see, e.g.,^15−18,23,24,27,31,36,53−70^ and references therein). Furthermore, note that for any α, the difference of the subdensities equals
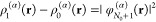
6namely, the highest (partially) occupied KS orbital (HOMO).

Unlike *n*_*p*_(**r**), the quantities  depend on α, because  do, and therefore the KS density (eq 4) has both an *explicit*, linear dependence on α and an implicit one, via  But since the KS density must equal the interacting one, , for any α, this introduces a constraint: ρ_0_ and ρ_1_ have such an α-dependence that their linear combination is exactly linear in α. This is what we explore in the following sections.

### 2.2 Taylor Expansion at α = 0^+^ and α = 1^–^

As a first step, we perform the regular Taylor expansion for both , in terms of α, at α = 0^+^. Expressing
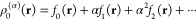
7and
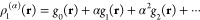
8where
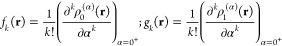
9we construct the KS density of eq 4 as  and equate it to the interacting density of eq 2, *n*_0_(**r**) + α(*n*_1_(**r**) – *n*_0_(**r**)), order-by-order. We assume here that all the derivatives with respect to α exist (see, however, Section 3.3 for a contrary numerical example).

For the zeroth order we get, unsurprisingly, *f*_0_(**r**) = *n*_0_(**r**). For the first order we obtain
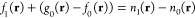
10Then we recall that , and therefore
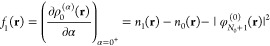
11*f*_1_(**r**) can be viewed as the difference between the Fukui function for electron addition in the *N*_0_-system, (*n*_1_(**r**) – *n*_0_(**r**)), and its crude, but common approximation, the LUMO,  (cf. ref (36)). For all the higher orders, the piecewise-linearity requirement for *n*(**r**) yields the following relations for the Taylor coefficients:
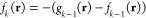
12with *k* ≥ 2. Using eq 6, the latter statement can also be expressed as
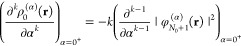
13for *k* ≥ 2. It follows from either eq 12 or 13 that if we knew the expansion of, say,  in α, we could get  and of course , and vice versa.

Notably, at first sight the convergence of the series in eq 8 is not guaranteed for arbitrary α. However, for α = 1, we get , and using eqs 10 and 12, it can be expressed as ρ_1_^(1)^(**r**) = *n*_1_(**r**) − *f*_1_(**r**) + , which equals *n*_1_(**r**), as required.

Furthermore, note that
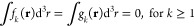
14This follows from the fact that  (left-hand side of eqs 7 and 8), and *f*_0_(**r**) and *g*_0_(**r**) (which have been identified above) integrate to *N*_0_ and *N*_0_ + 1, respectively.

A Taylor expansion around α = 1^–^ is analogous, and is easier to perform defining α′ = 1 – α. It contributes the following two results:
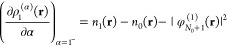
15and
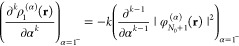
16for *k* ≥ 2 (cf. eqs 11 and 13). In eq 15 the derivative ∂ρ_1_/∂α can be viewed as the difference between the Fukui function for electron removal in the (*N*_0_ + 1)-system and the HOMO of that system,  (cf. eq 11).

### 2.3 Two-Point Taylor Expansion (2pTE)

A much less-known expansion technique is a two-point Taylor expansion (2pTE), which expresses a general function *y*(α), using information about this function and its derivatives in two preselected points^71^ (see also^72−76^). This method seems most relevant in our case, as we have information about the system at the edges of the α-range: at α = 0^+^ and at α = 1^–^.

Adapting the results of ref (71), we express a sufficiently differentiable function *y*(α) in a 2pTE as
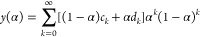
17The coefficients *c*_*k*_ and *d*_*k*_ are determined recursively, assuming knowledge of the *k*th derivatives of *y*(α) at α = 0^+^ and α = 1^–^:
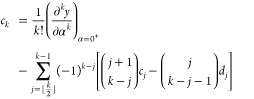
18
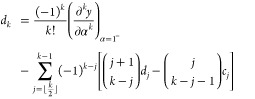
19The coefficients *c*_*k*_ are closely related to the derivatives’ values at 0^+^, while *d*_*k*_ are related to 1^–^. In particular,
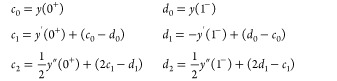
20and so on.

As an alternative to eq 17, one can also choose the following expansion:
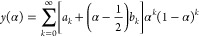
21where the *a*-terms are symmetric with respect to α =  and the *b*-terms are antisymmetric. These two expansions are equivalent, with linear relations between their coefficients.

We perform 2pTEs for both subdensities ρ_*p*_:
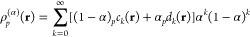
22From eq 20 we realize that
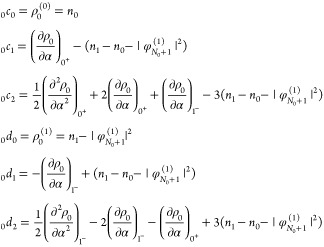
23and
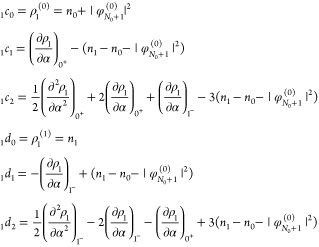
24(the **r**-dependence of all quantities has been suppressed here for brevity). Since _*p*_*c*_0_ and _*p*_*d*_0_ are identified with quantities that integrate to *N*_0_ + *p*, we conclude that
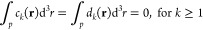
25We stress that all the expressions presented in this section so far are the result of a 2pTE, and the piecewise-linearity requirement has not been applied here yet.

Next, to apply piecewise-linearity, we construct the KS density (eq 4) using the expansions in eq 22, and utilizing the identities
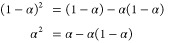
26find that
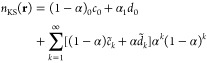
27where
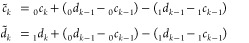
28for *k* ≥ 1. Requiring piecewise-linearity, namely equating the expression in eq 27 to eq 2 order-by-order, we find that _0_*c*_0_(**r**) = *n*_0_(**r**) and _1_*d*_0_(**r**) = *n*_1_(**r**), in agreement with eqs 23 and 24. Furthermore, *c̃*_*k*_(**r**) and *d̃*_*k*_(**r**) must vanish, which means that
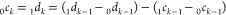
29These are the two restrictions on the 2pTE of the subdensities ρ_*p*_: (i) the *c*-coefficients of  equal the *d*-coefficients of , for *k* ≥ 1; (ii) two coefficients of order *k* are set by coefficients of order *k* – 1 (if the latter are known). The other two coefficients, _1_*c*_*k*_ and _0_*d*_*k*_, remain, however, undetermined.

The right-hand side of eq 29, which we denote *Q*_*k*–1_, can be expressed recursively, using eqs 17 and 6:
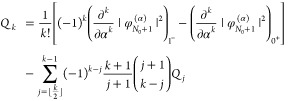
30

Applying the piecewise-linearity restriction, eq 29, to the 2pTE coefficients (eqs 23, 24), we obtain
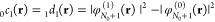
31These terms can be viewed as relaxation terms, as they stem from the differences in the HOMO orbital energies at the edges of the α-range. In passing, we derived again eqs 11 and 15.^77^ Using the latter and recalling eq 6, we can express the remaining first-order coefficients as
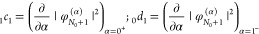
32The above expressions do not define _1_*c*_1_ and _0_*d*_1_, but just relate them to derivatives of the HOMO.

Next, for the second-order terms, we find that
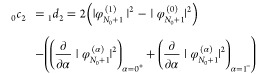
33and
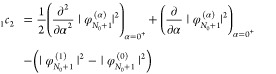
34
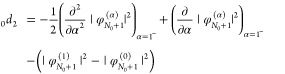
35Finally, in the derivation process we find another relation for the first-order terms:
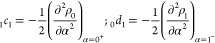
36Turning our attention to the HOMO, we perform a 2pTE,
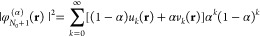
37and realize from eq 6 that *u*_*k*_ = _1_*c*_*k*_ – _0_*c*_*k*_ and *v*_*k*_ = _1_*d*_*k*_ – _0_*d*_*k*_. However, applying here the restrictions we derived for ρ_*p*_ (i.e., eq 29 in conjunction with eqs 23 and 24), we find that no restrictions emerge for *u*_*k*_ and *v*_*k*_: we obtain the same expressions as in eq 20, when *y* is substituted by |φ_*N*_0_+1_|^2^. Therefore, surprisingly, the piecewise-linearity requirement for the density does not directly constrain the HOMO orbital density. This does not mean that the HOMO will not change if we impose piecewise-linearity, but just that we did not establish any relation between the expansion coefficients of the HOMO, or between the latter and coefficients of other quantities.

### 2.4 Approximate Regimes for the HOMO

As mentioned above, the piecewise-linearity requirement for the density does not constrain the HOMO. Furthermore, we note that the 2pTE coefficients of  can be expressed via the HOMO orbital density , and its derivatives, at 0^+^ and 1^–^. Therefore, if we assume (or numerically establish) a certain approximate α-dependence for the HOMO, it will dictate the α-dependence of ρ_*p*_. In this section, we examine four such α-regimes. In each case, we discuss the implications of a given approximation for the orbitals, before and after the piecewise-linearity requirement is imposed.

#### (a) Frozen Approximation

The standard frozen approximation assumes no relaxation of orbitals upon variation of the electron number. In other words, all orbitals are α-independent. For the HOMO, . Furthermore, , is α-independent, and similarly is . It then follows that
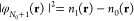
38namely the Fukui function for electron removal in the (*N*_0_ + 1)-system equals the HOMO of that system. Similarly, the Fukui function for electron addition in the *N*_0_-system equals the LUMO of that system.

In terms of eq 37, the frozen approximation means that *u*_0_(**r**) = *v*_0_(**r**) = |φ_*N*_0_+1_(**r**)|^2^ and that for *k* ≥ 1, *u*_*k*_(**r**) = *v*_*k*_(**r**) = 0. As to the 2pTE coefficients of ρ_*p*_ in eqs 23 and 24, we realize that _0_*c*_0_(**r**) = _0_*d*_0_(**r**) = *n*_0_(**r**), _1_*c*_0_(**r**) = _1_*d*_0_(**r**) = *n*_1_(**r**), and that _0_*c*_1_(**r**) = _0_*d*_1_(**r**) = _1_*c*_1_(**r**) = _1_*d*_1_(**r**) = 0, and similarly for higher coefficients. Remarkably, the piecewise-linearity requirement for the density becomes trivial: in the crude frozen approximation the ensemble density is piecewise-linear by construction.

#### (a′) Partially Frozen Approximation

Here we consider a weaker approximation, namely that only the HOMO is frozen, whereas other orbitals may depend on α.

In terms of eq 37, the statements *u*_0_(**r**) = *v*_0_(**r**) = |φ_*N*_0_+1_(**r**)|^2^ and *u*_*k*_(**r**) = *v*_*k*_(**r**) = 0, for *k* ≥ 1, still hold, as in (a). However, eq 38 does not hold anymore. As to the coefficients in eqs 23 and 24, we find that _0_*c*_0_(**r**) = *n*_0_(**r**), _0_*d*_0_(**r**) = *n*_1_(**r**) – |φ_*N*_0_+1_(**r**)|^2^ (but _0_*d*_0_(**r**) ≠ _0_*c*_0_(**r**) anymore). Similarly, _1_*c*_0_(**r**) = *n*_0_(**r**) + |φ_*N*_0_+1_(**r**)|^2^ and _1_*d*_0_(**r**) = *n*_1_(**r**); _1_*c*_0_(**r**) ≠ _1_*d*_0_(**r**). Keeping in mind that |φ_*N*_0_+1_(**r**)|^2^ is α-independent here and using eq 6, we find that in this regime ∂ρ_0_/∂α = ∂ρ_1_/∂α. Therefore, _0_*c*_1_(**r**) = _1_*c*_1_(**r**) and _0_*d*_1_(**r**) = _1_*d*_1_(**r**), but they are not all equal, as in (a) before. Similarly, _0_*c*_2_(**r**) = _1_*c*_2_(**r**) and _0_*d*_2_(**r**) = _1_*d*_2_(**r**), etc. The above statements do not automatically make the ensemble density *n*(**r**) piecewise-linear.

Requiring piecewise-linearity in this regime introduces eqs 31 and 32. This results in the requirement
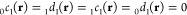
39and similarly for higher orders. As a result, the subdensities are piecewise-linear:
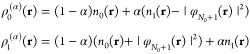
40and so is the ensemble density, *n*(**r**).

#### (b) Linear Approximation

Making one step beyond the frozen approximation (a), let us assume that all orbitals can relax, but their dependence on α is linear:
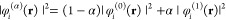
41As a result,  are linear, as well:
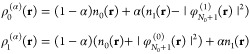
42(note the difference vs eq 40). Interestingly, the ensemble density does not come out linear:
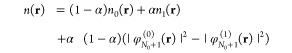
43It includes a symmetric parabolic term, which vanishes only if , i.e., if the HOMO is frozen, in agreement with eq 31.

#### (b′) Partially Linear Approximation

By analogy with (a′), let us assume that the HOMO relaxes linearly, while relaxation of other orbital densities may include also nonlinear terms.

In terms of eq 37, ,  (*u*_0_(**r**) ≠ *v*_0_(**r**)) and *u*_*k*_(**r**) = *v*_*k*_(**r**) = 0, for *k* ≥ 1. Furthermore,
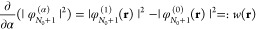
44is independent of α.

As to the coefficients in eqs 23 and 24, we find that _0_*c*_0_(**r**) = *n*_0_(**r**), _0_*d*_0_(**r**) = *n*_1_(**r**) − |φ_*N*_0_+1_^(1)^(**r**)|^2^,  and _1_*d*_0_(**r**) = *n*_1_(**r**) – all different from each other. Furthermore, even the differences (_1_*c*_0_ – _0_*c*_0_) and (_1_*d*_0_ – _0_*d*_0_) are not equal.

From eq 44 we realize that in this regime ∂ρ_1_/∂α = ∂ρ_0_/∂α + *w*(**r**) and ∂^*k*^ρ_1_/∂α^*k*^ = ∂^*k*^ρ_0_/∂α^*k*^, for *k* ≥ 2. Therefore,  and . For the second-order coefficients, we find that _0_*c*_2_(**r**) = _1_*c*_2_(**r**) and _0_*d*_2_(**r**) = _1_*d*_2_(**r**), as in (a*′*).

If we require piecewise-linearity, we find that _0_*c*_1_ = _1_*c*_1_ = _0_*d*_1_ = _1_*d*_1_ = *w*(**r**) and _0_*c*_2_ = _1_*c*_2_ = _0_*d*_2_ = _1_*d*_2_ = 0. The subdensities then equal
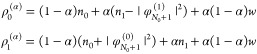
45having a nonvanishing parabolic term, symmetric with respect to α = ; no antisymmetric term appears.

In summary, comparing (a*′*) and (b*′*), we find that an α-independent HOMO implies linear ρ_*p*_(**r**), while a linear HOMO implies a parabolically dependent ρ_*p*_(**r**), and so on. This is somewhat reminiscent of the relation between the HOMO energy level,  and the derivative of the total energy, *E*, with respect to *N* (although no exact relation, which connects the HOMO orbital density to the derivative of ρ_*p*_(**r**) versus α is known).

## 3 Numerical Investigation

### 3.1 Technical Details

To illustrate our analytical findings numerically, DFT calculations for the atoms of H, Li, Na, K, B, Al, Cu, Ag and Au, with the number of electrons varying between that of a neutral atom and that of the first ion, were performed. Furthermore, to deeper understand the results for Li, calculations for a series of Li-like ions, namely Be^+^, B^2+^, C^3+^, N^4+^ and Al^10+^, were carried out. In all these ions, like in Li, the number of electrons varied between 2 and 3. For simplicity, at this stage only spin-unpolarized calculations were considered. For the xc approximation, the commonly used LDA^38^ and PBE GGA functionals,^78^ as well as invLDA and invPBE,^37^ were explored.

All calculations were carried out with the ORCHID atomic spherical DFT code.^79,80^ In addition, the 2pTEc code was written to extract and analyze the two-point Taylor expansion coefficients. Both codes are available online^81^

A logarithmic grid with *N* = 16.000 points was used on the interval [*r*_min_, *r*_max_], where *r*_min_ = e^–13^/*Z* Bohr, with *Z* being the atomic number, and *r*_max_ = 35 Bohr. To maintain high accuracy in the coefficients, the convergence of the SCF cycle was brought to its numerical limit, which resulted in energy differences between the two last iterations being below Δ_*E*_ = 4 × 10^–8^ Hartree for Cu, 10^–8^ Hartree for Al and Au, 10^–9^ Hartree for Na, K and Ag, 10^–10^ Hartree for B, B^2+^, C^3+^, N^4+^, Al^10+^, and 10^–11^ Hartree for H, Li and Be^+^. In terms of the density convergence, the radial integral Δ_*n*_:= |∫ d*rn*(*r*) – *n*^*f*^(*r*)|, where *n*(*r*) and *n*^*f*^(*r*) are the densities of the two last iterations, was kept below 10^–7^ a.u. for Cu, 10^–8^ a.u. for Au, 10^–9^ a.u. for Na, K and Ag, 10^–10^ a.u. for Al, 10^–10^ a.u. for B^2+^, C^3+^, N^4+^, Al^10+^ and 10^–12^ a.u. for H, Li, B and Be^+^. For the inversion calculations, it was required that the difference between the resultant and the target ensemble densities does not exceed 10^–4^ a.u., at any point in space.

To accurately obtain the expansion coefficients, the following values of α were considered. In addition to α = 0.1, 0.2, ···, 0.9, α = 10^–*n*^ was added, to obtain the limit α → 0^+^, with *n* = 1, 2, 3, 4 at least; higher values of *n* were considered if needed for convergence. Similarly, the values α = 1–10^–*n*^ were added to obtain the limit α → 1^–^.

In the following sections, we analyze not the (sub-) densities themselves, but the latter multiplied by 4π*r*^2^, versus the distance from the nucleus, *r*, as is common in atomic calculations. All properties discussed above still apply, of course. Hartree atomic units are used throughout.

### 3.2 Typical Example: The Ag Atom

The Ag atom is presented below, as a typical example. Similar results were obtained also for B, Al, Cu and Au (see the Supporting Information (SI) for details).

**Figure 1:**
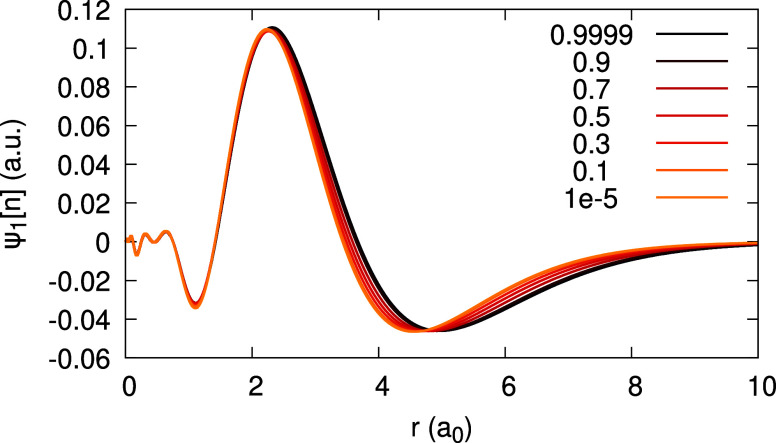
Quantity ψ_1_[*n*](*r*;α) (defined in text), obtained for the Ag atom, within the LDA, for different values of α (see Legend).

The deviation of the density from piecewise-linearity is illustrated in Figure 1, plotting the following quantity:
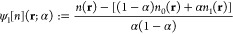
46for the LDA. In words, ψ_1_[*f*](**r**;α) is the difference between a quantity *f* (in our case, this is the density *n*), obtained for a fractional electron number *N* = *N*_0_ + α, and the linear combination of the same quantity at the edges of the α-range, at α = 0^+^ and 1^–^, all this divided by the factor α (1 – α). The limits of ψ_1_[*n*] yield *c̃*_1_(**r**) = lim_α→0^+^_ ψ_1_[*n*](**r**; α) and *d̃*_1_(**r**) = lim_α→1^–^_ ψ_1_[*n*](**r**;α).

**Figure 2:**
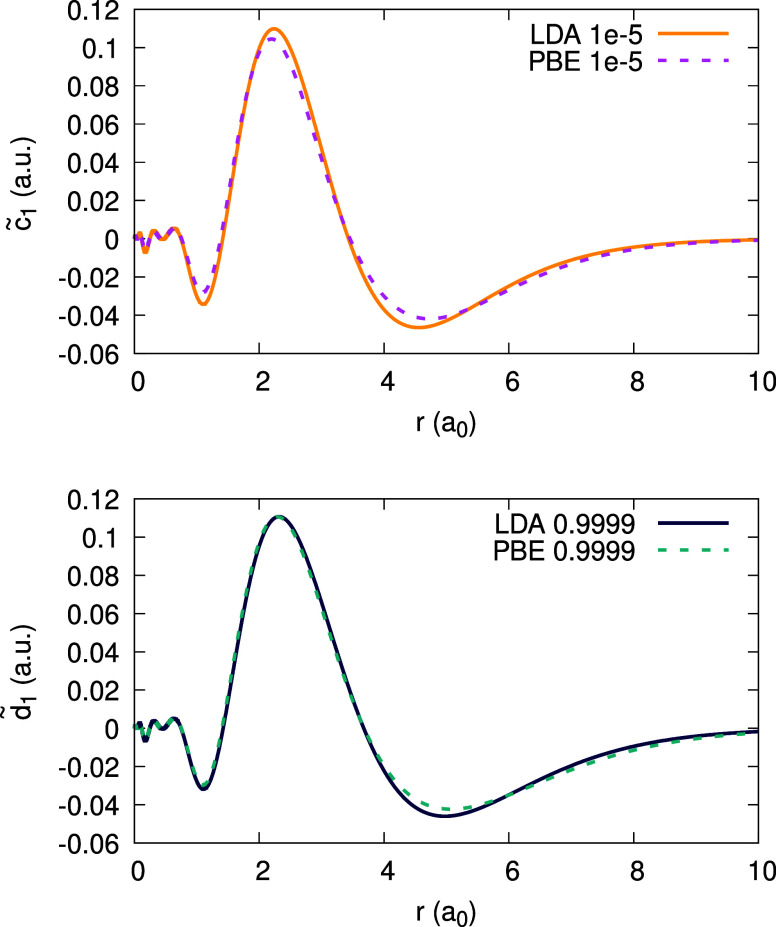
Expansion coefficients *c̃*_1_(*r*) (top) and *d̃*_1_(*r*) (bottom), obtained for the Ag atom, within the LDA and PBE (see Legend; the number to the right of the xc abbreviation is a value of α for which the coefficient function is converged).

From eq 27, we realize that for the exact functional ψ_1_[*n*](**r**;α) has to be zero, and for approximations it measures the deviation from piecewise-linearity. The coefficients *c̃*_1_(**r**) and *d̃*_1_(**r**) are depicted in Figure 2, for the LDA and PBE. The difference between LDA and PBE results is recognizable, but insignificant.

For invLDA and invPBE, where piecewise-linearity of the density is enforced, the coefficients *c̃*_1_(**r**) and *d̃*_1_(**r**) vanish: for the latter functionals, they differ from 0 by no more than 10^–4^ a.u., for any *r* and α, in agreement with the requirement introduced in Section 3.1 (see Figure 1 in the SI). The integrals ∫ *c̃*_1_(**r**) d^3^*r* and ∫ *d̃*_1_(**r**) d^3^*r* deviate from 0 by no more than 10^–9^, for all approximations, as expected (cf. eq 25).

Higher coefficients of the density, *c̃*_*k*_(**r**) and *d̃*_*k*_(**r**), can be obtained in a similar manner, analyzing
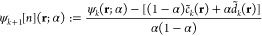
47for *k* ≥ 1. The numerical effort required to converge these results is much higher, though. Yet, their contribution to the density expansion of Ag is negligible: *c̃*_2_ and *d̃*_2_ are lower than *c̃*_1_ and *d̃*_1_ by an order of magnitude, at least. Another way to estimate the (in)significance of these terms is by calculating ∫ (*c̃*_*k*_(**r**))^2^ d^3^*r* and ∫ (*d̃*_*k*_(**r**))^2^ d^3^*r*. Whereas the integral of *c̃*_*k*_(**r**) and of *d̃*_*k*_(**r**) has to be zero, both for the exact and approximate cases, the integral of their squares is an indicator of how much these functions differ from the zero function. For *k* = 2, the above integrals are lower than 5 × 10^–6^ a.u., which is 2–3 orders of magnitude lower than for *c̃*_1_(**r**) and *d̃*_1_(**r**). Therefore, we conclude that for Ag these higher orders can safely be omitted.

**Figure 3:**
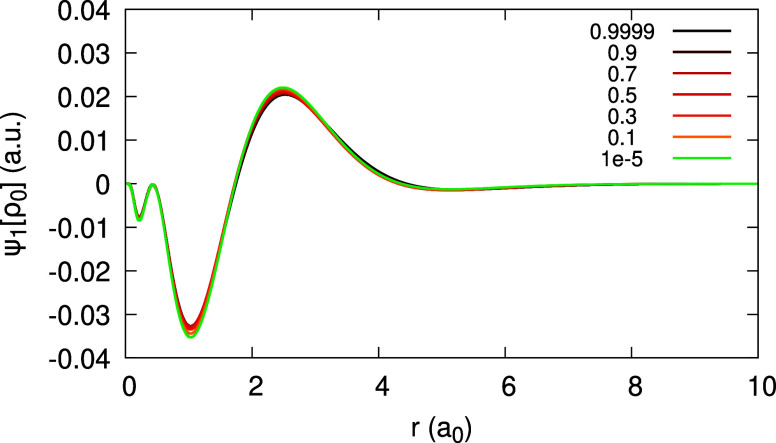
Quantity ψ_1_[ρ_0_](*r*;α) (defined in text), obtained for the Ag atom, within the LDA, for different values of α (see Legend).

Moving on to the subdensities ρ_0_ and ρ_1_, we analyze them in a similar manner. Figure 3 shows the function ψ_1_[ρ_0_](**r**;α) for Ag obtained with the LDA. We find that ψ_1_[ρ_0_] is of modest magnitude of 0.04 au and is barely changing with α over the entire α-range. Similar behavior of ψ_1_[ρ_0_] we found also when using PBE.

**Figure 4:**
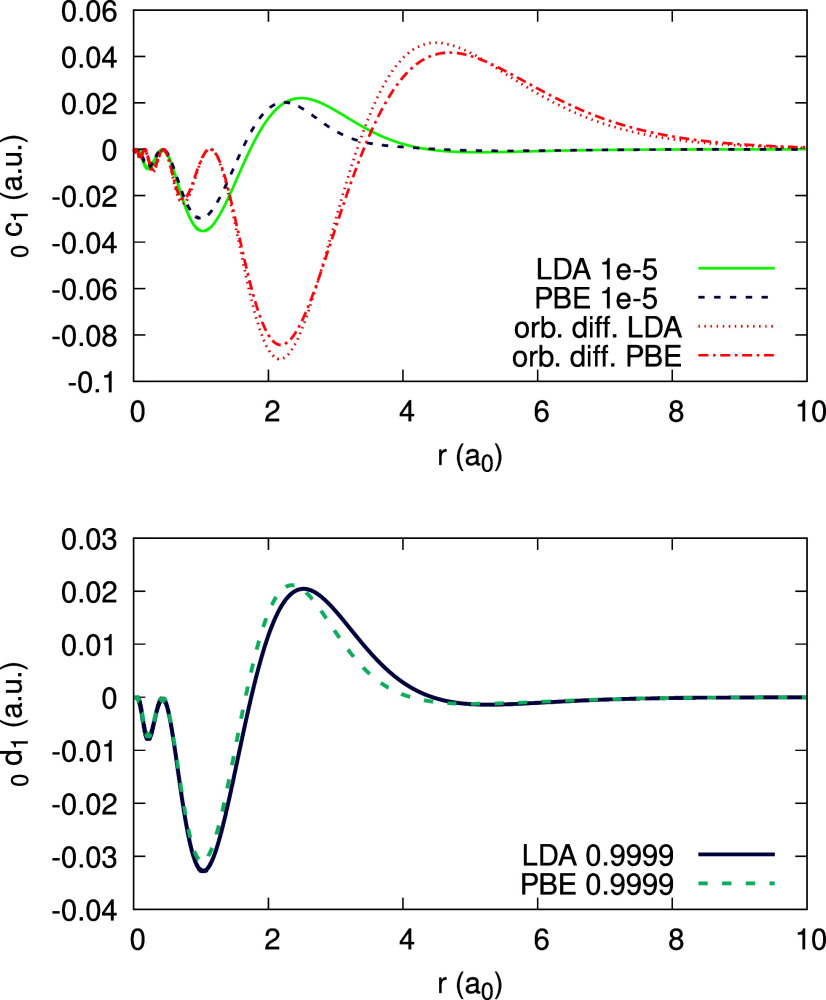
Expansion coefficients _0_*c*_1_(*r*) (top) and _0_*d*_1_(*r*) (bottom), obtained for the Ag atom, within the LDA and PBE (see Legend; the number to the right of the xc abbreviation is a value of α for which the coefficient function is converged). Orbital difference (orb. diff. in the Legend) from the rhs of eq 31 is shown along _0_*c*_1_(*r*) for comparison, for each functional.

From the data on ψ_1_[ρ_0_], the coefficients _0_*c*_1_(**r**) and _0_*d*_1_(**r**) can be deduced; they are shown in Figure 4, using the LDA and the PBE functionals, for each coefficient. In addition, recalling eq 31, the orbital difference , which should equal _0_*c*_1_(**r**), is plotted for comparison. We observe an enormous discrepancy between this difference and _0_*c*_1_(**r**). This should not surprise us: both the LDA and PBE are far from satisfying the piecewise-linearity property for the density.

**Figure 5:**
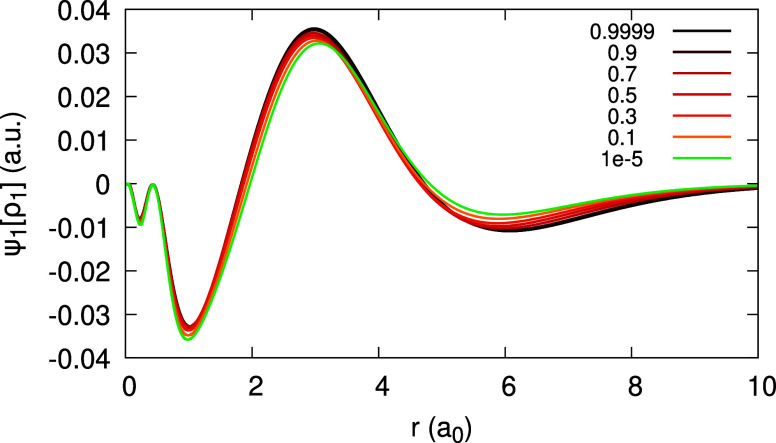
Quantity ψ_1_[ρ_1_](*r*;α) (defined in text), obtained for the Ag atom, within the LDA, for different values of α (see Legend).

**Figure 6:**
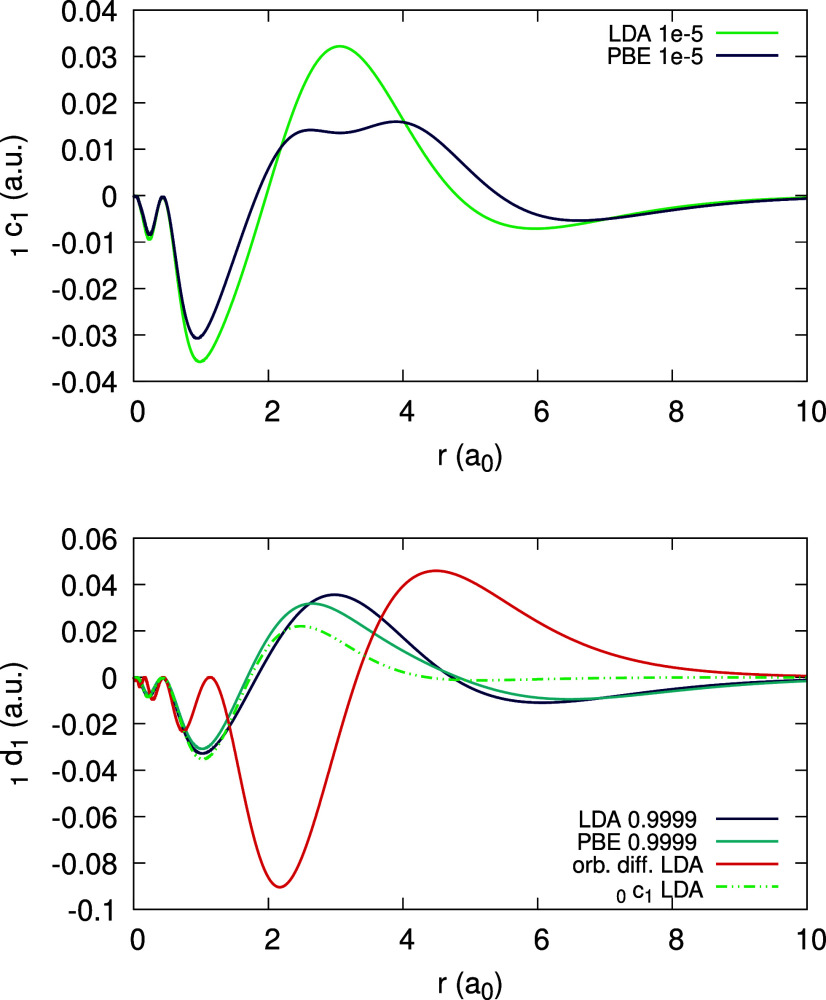
Expansion coefficients _1_*c*_1_(*r*) (top) and _1_*d*_1_(*r*) (bottom), obtained for the Ag atom, within the LDA and PBE (see Legend; the number to the right of the xc abbreviation is a value of α for which the coefficient function is converged). Orbital difference (orb. diff. in the Legend) at the rhs of eq 31 and the coefficient _0_*c*_1_(*r*) are shown for _1_*d*_1_, for comparison, for the LDA.

Next, the quantity ψ_1_[ρ_1_](**r**;α) is plotted in Figure 5 for Ag within the LDA. We observe a qualitatively similar behavior to that of ψ_1_[ρ_0_](**r**;α) in Figure 3, again with only a mild change with α. The corresponding coefficients _1_*c*_1_(**r**) and _1_*d*_1_(**r**) appear in Figure 6, for the LDA and the PBE functionals. For _1_*c*_1_ we find a more significant discrepancy between the LDA and PBE results, comparing to all other coefficients. Therefore, the numerical convergence of both curves has been carefully checked: results with α = 10^–3^ and 10^–4^ coincide with the curves depicted. Moreover, we find that _1_*d*_1_ significantly varies from both _0_*c*_1_ and the rhs of eq 31, whereas they should all coincide.

**Figure 7:**
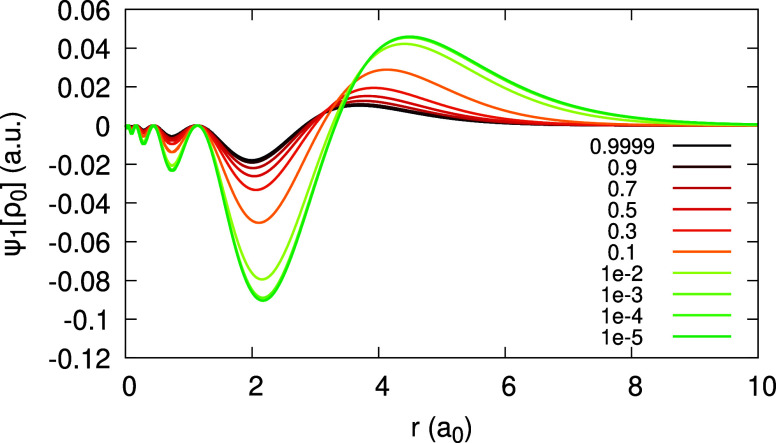
Quantity ψ_1_[ρ_0_](*r*; α) (defined in text), obtained for the Ag atom, within the invLDA, for different values of α (see Legend).

**Figure 8:**
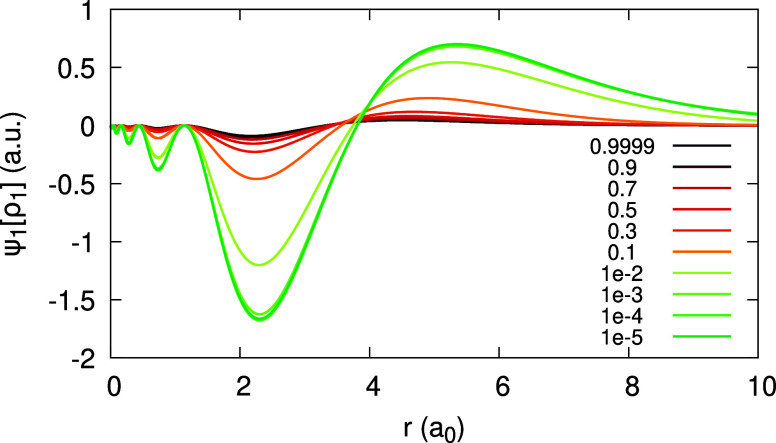
Quantity ψ_1_[ρ_1_](*r*; α) (defined in text), obtained for the Ag atom, within the invLDA, for different values of α (see Legend).

Finally, we obtain ψ_1_[ρ_0_](**r**;α) and ψ_1_[ρ_1_](**r**;α) for invLDA and invPBE and observe the effect of enforcement of piecewise-linearity in the density on our results. We focus on invLDA, as the difference between invLDA and invPBE remains small. Figures 7 and 8 show, respectively, plots for ψ_1_[ρ_0_](**r**;α) and ψ_1_[ρ_1_](**r**;α), within invLDA. The difference with respect to LDA results is dramatic: first, for both quantities the dependence on α is substantial, with particularly large differences occurring as α → 0^+^. Second, whereas the magnitude of ψ_1_[ρ_0_] in invLDA is comparable to that of LDA, for ψ_1_[ρ_1_] the magnitude is more than 20 times larger.

**Figure 9:**
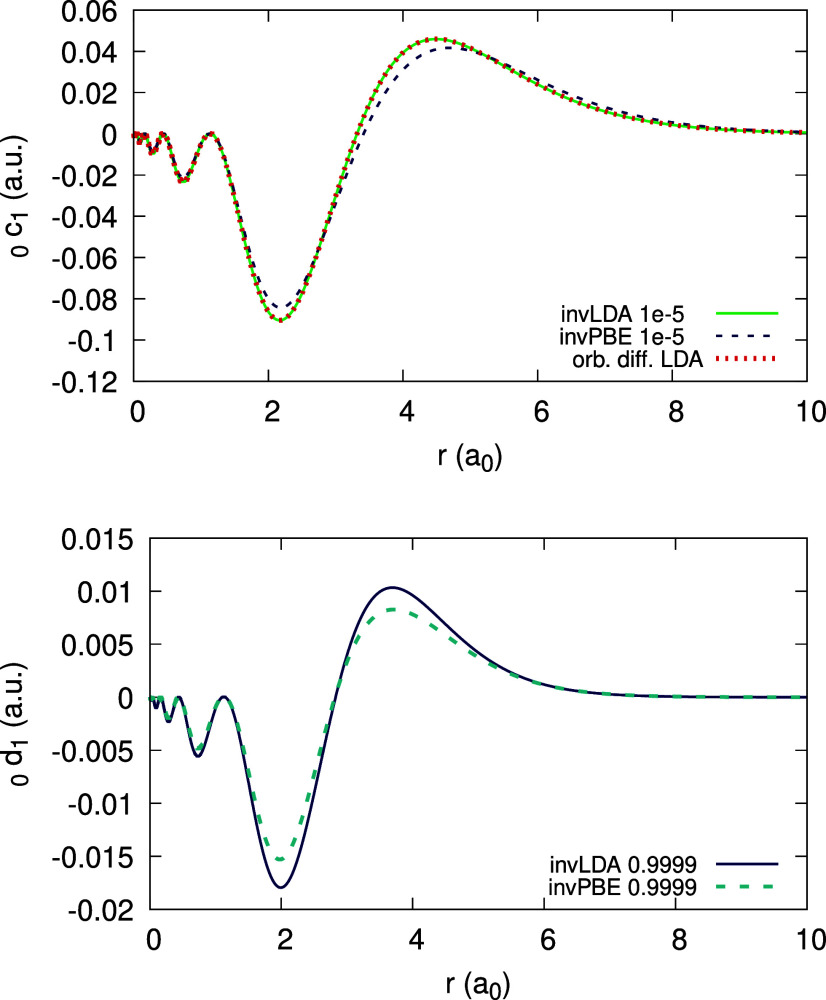
Expansion coefficients _0_*c*_1_(*r*) (top) and _0_*d*_1_(*r*) (bottom), obtained for the Ag atom, within the invLDA and invPBE (see Legend; the number to the right of the xc abbreviation is a value of α for which the coefficient function is converged). Orbital difference (orb. diff. in the Legend) from the rhs of eq 31 is shown for comparison in the top panel (overlaps with the _0_*c*_1_(*r*) invLDA curve).

**Figure 10:**
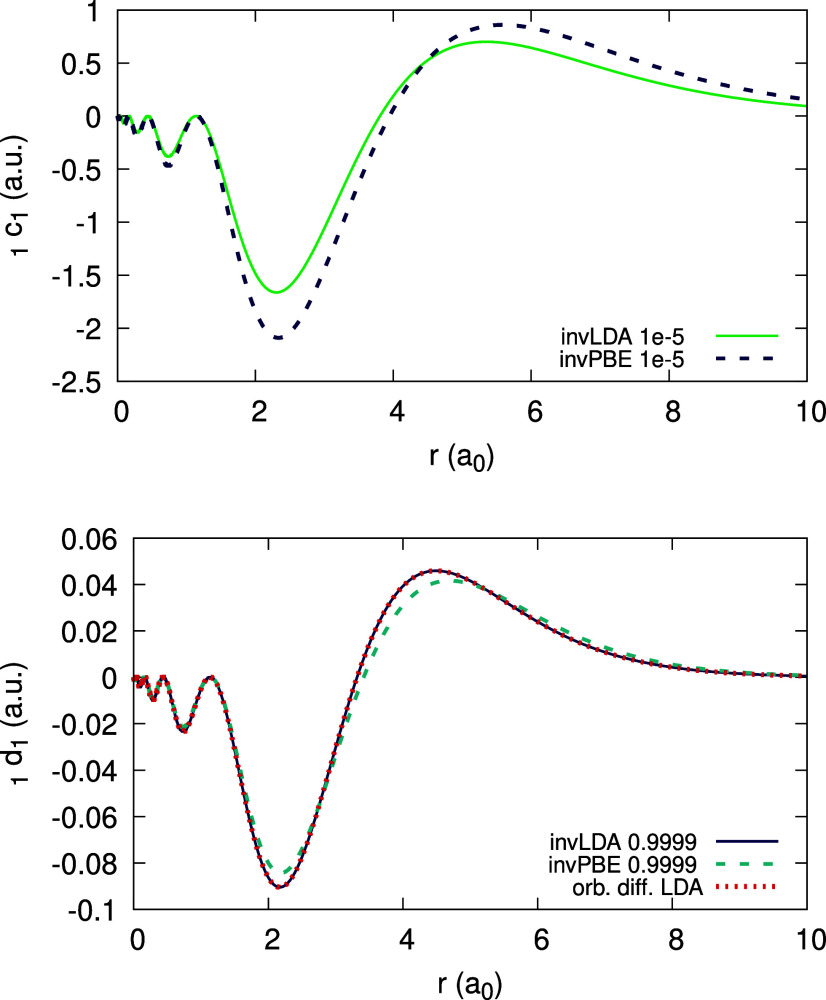
Expansion coefficients _1_*c*_1_(*r*) (top) and _1_*d*_1_(*r*) (bottom), obtained for the Ag atom, within the invLDA and invPBE (see Legend; the number to the right of the xc abbreviation is a value of α for which the coefficient function is converged). Orbital difference (orb. diff. in the Legend) at the rhs of eq 31 is shown for comparison in the bottom panel (overlaps with the _1_*d*_1_(*r*) invLDA curve).

The expansion coefficients _0_*c*_1_(**r**), _0_*d*_1_(**r**), _1_*c*_1_(**r**) and _1_*d*_1_(**r**) are presented in Figures 9 and 10. In contrast to the LDA and PBE, we find close correspondence between _0_*c*_1_ and _1_*d*_1_ and the rhs of eq 31: the curves fully overlap in both figures. This is a direct result of enforcing piecewise-linearity of the density. Furthermore, we observe that invPBE results show only a small difference versus invLDA.

**Figure 11:**
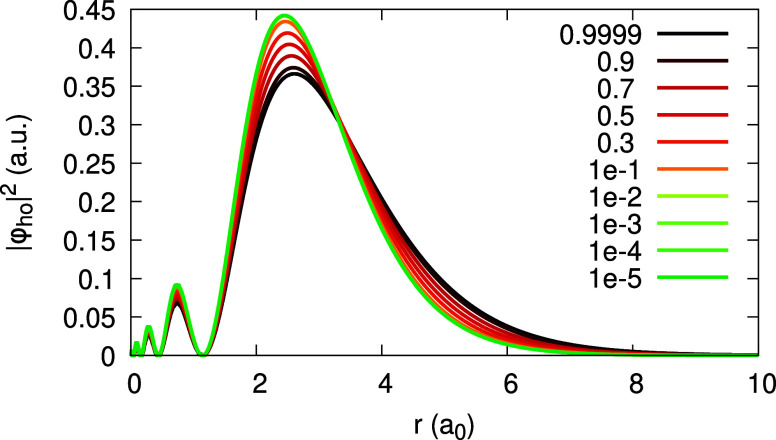
HOMO orbital density for the Ag atom, obtained within the LDA, for various values of α (see Legend).

**Figure 12:**
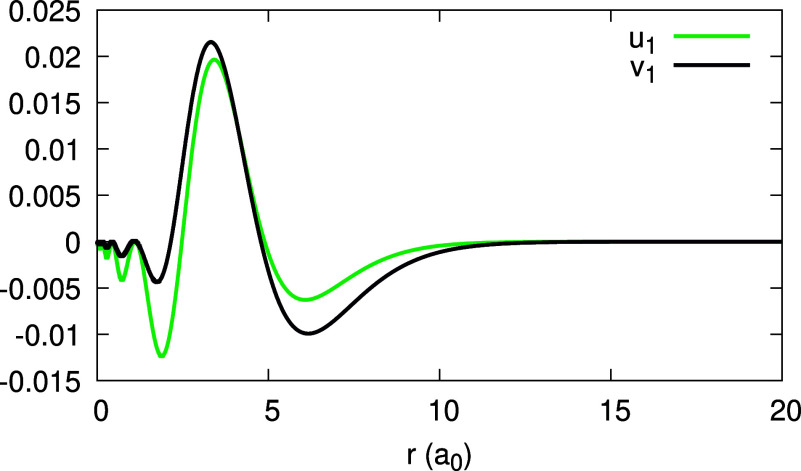
Expansion coefficients *u*_1_(*r*) and *v*_1_(*r*), obtained for the Ag atom with the LDA (see Legend).

**Figure 13:**
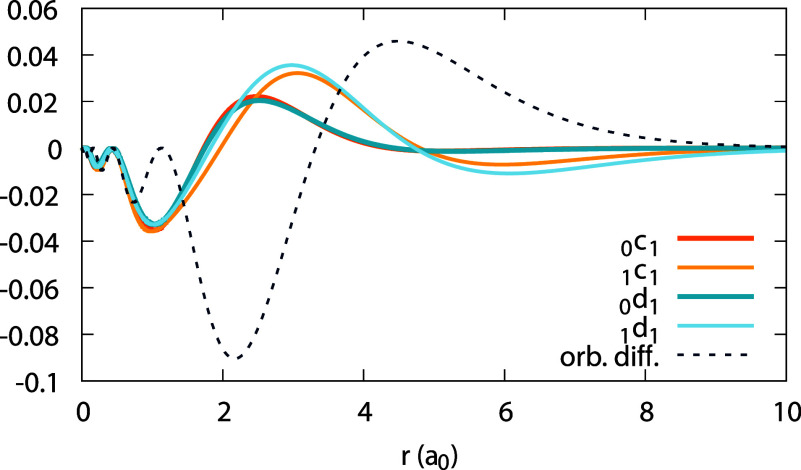
Expansion coefficients _0_*c*_1_(*r*), _1_*c*_1_(*r*), _0_*d*_1_(*r*), and _1_*d*_1_(*r*) for the Ag atom obtained with the LDA (see Legend), along with the orbital difference *w*(*r*) of eq 44.

We conclude this section by analyzing the HOMO orbital density, bearing in mind the various regimes we detailed in Section 2.4. The dependence of the HOMO on α is illustrated for the LDA in Figure 11. Clearly, the HOMO is not frozen: it gradually changes with α, and the change appears roughly linear. The expansion coefficients, *u*_1_(**r**) and *v*_1_(**r**), presented in Figure 12, although not being zero, as required in Regime (b*′*) of Section 2.4, are lower by 1 order of magnitude comparing to *u*_0_(**r**) and *v*_0_(**r**) (i.e., the HOMO density of Figure 11). From Figure 13 we learn that the coefficients _0_*c*_1_ and _1_*c*_1_ roughly equal each other closer to the nucleus, grow apart at *r* ∼ 2–4 Bohr, and then coincide asymptotically. Similar is the situation for _0_*d*_1_ and _1_*d*_1_. All these coefficients are of course far from *w*(**r**) of eq 44, for the LDA. Therefore, we conclude that among the regimes presented in Section 2.4, the HOMO for Ag with LDA is closer to Regime (b*′*), although a nonlinear α-dependence of the HOMO is present.

**Figure 14:**
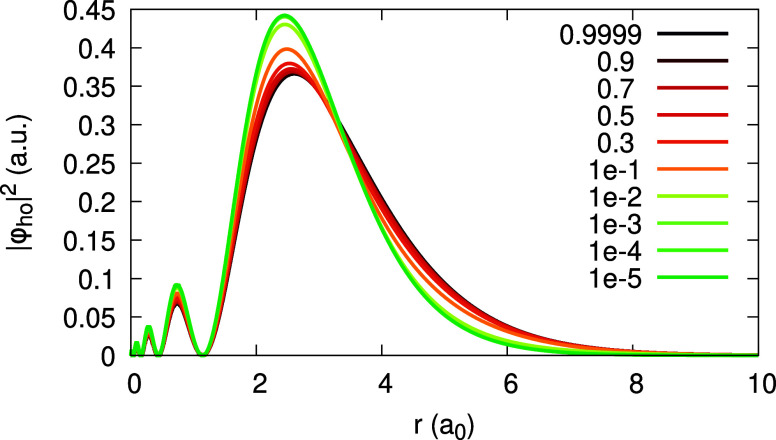
HOMO orbital density for the Ag atom, obtained within invLDA, for various values of α (see Legend).

**Figure 15:**
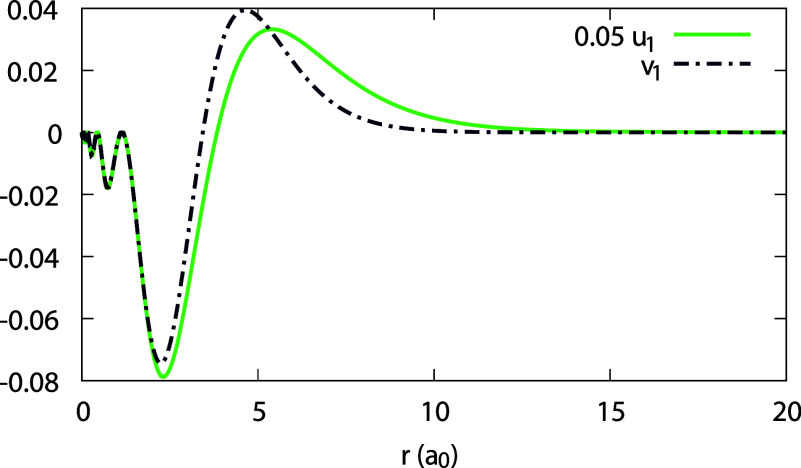
(Scaled) expansion coefficients 0.05 *u*_1_(*r*) and *v*_1_(*r*), obtained for the Ag atom with invLDA (see Legend).

**Figure 16:**
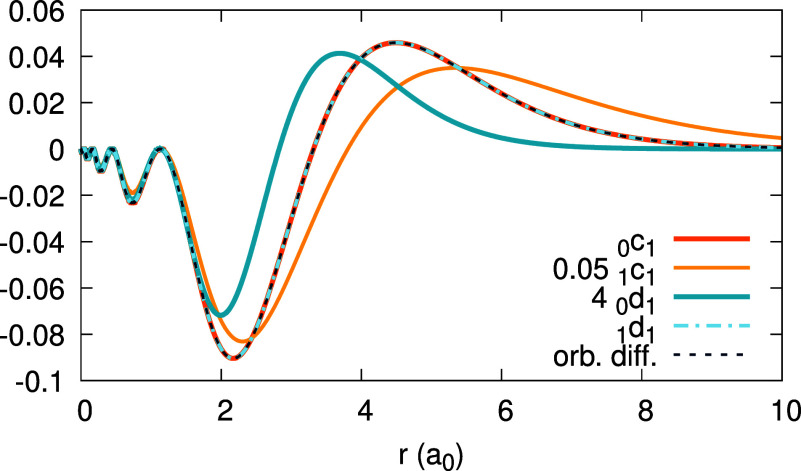
(Scaled) expansion coefficients _0_*c*_1_(*r*), 0.05 _1_*c*_1_(*r*), 4 _0_*d*_1_(*r*), and _1_*d*_1_(*r*) for the Ag atom obtained with invLDA (see Legend), along with the orbital difference *w*(*r*) of eq 44. Note that _0_*c*_1_(*r*), _1_*d*_1_(*r*) and *w*(*r*) coincide.

With invLDA, the situation is strikingly different, and lies beyond any of the aforementioned regimes for the HOMO. The α-dependence of the HOMO, presented in Figure 14, is rather uneven: it is almost frozen, for α ∈ [0.1, 1], but rapidly changes as α → 0^+^. As a result, the expansion coefficient *u*_1_(**r**) is absolutely dominant over *v*_1_(**r**) (note its scaling in Figure 15), and is by no means negligible comparing to *u*_0_(**r**) and *v*_0_(**r**). As to the coefficients _0_*c*_1_(**r**), _1_*c*_1_(**r**), _0_*d*_1_(**r**), and _1_*d*_1_(**r**), depicted in Figure 16, two of them, _0_*c*_1_(**r**) and _1_*d*_1_(**r**), equal each other and coincide with *w*(**r**), as we already found before. The dominant coefficient is _1_*c*_1_(**r**) – it is much larger than _0_*c*_1_(**r**); _0_*d*_1_(**r**) is the smallest. To conclude, enforcing piecewise-linearity with invLDA is clearly reflected in the fact that the expansion coefficients satisfy eq 31, but it does not result in a simple regime for the HOMO.

### 3.3 Atypical Example: The Li Atom

We now examine in detail the Li atom, which shows a fundamentally different behavior upon a two-point Taylor expansion.

**Figure 17:**
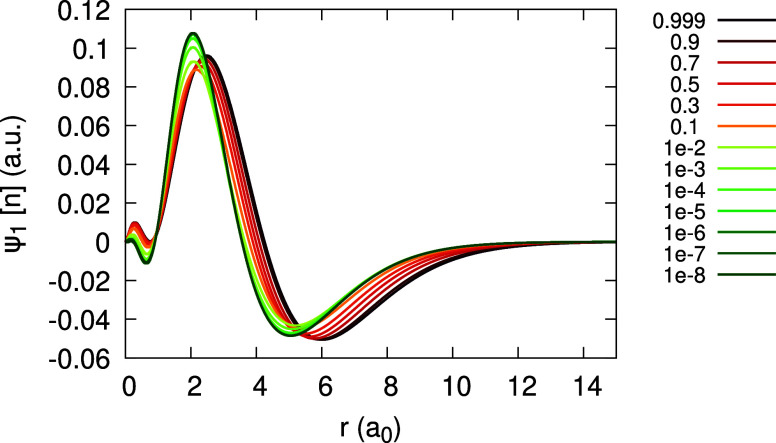
Quantity ψ_1_[*n*](*r*;α) (defined in text), obtained for the Li atom, within the LDA, for different values of α (see Legend).

The dependence of ψ_1_[*n*](**r**;α) on α, presented in Figure 17, is much more significant for Li with LDA than for Ag. To achieve convergence at α → 0^+^, α has been decreased to 10^–8^. Yet, convergence has been obtained both at 0^+^ and 1^–^, and *c̃*_1_(**r**) and *d̃*_1_(**r**) could be deduced (not plotted).

**Figure 18:**
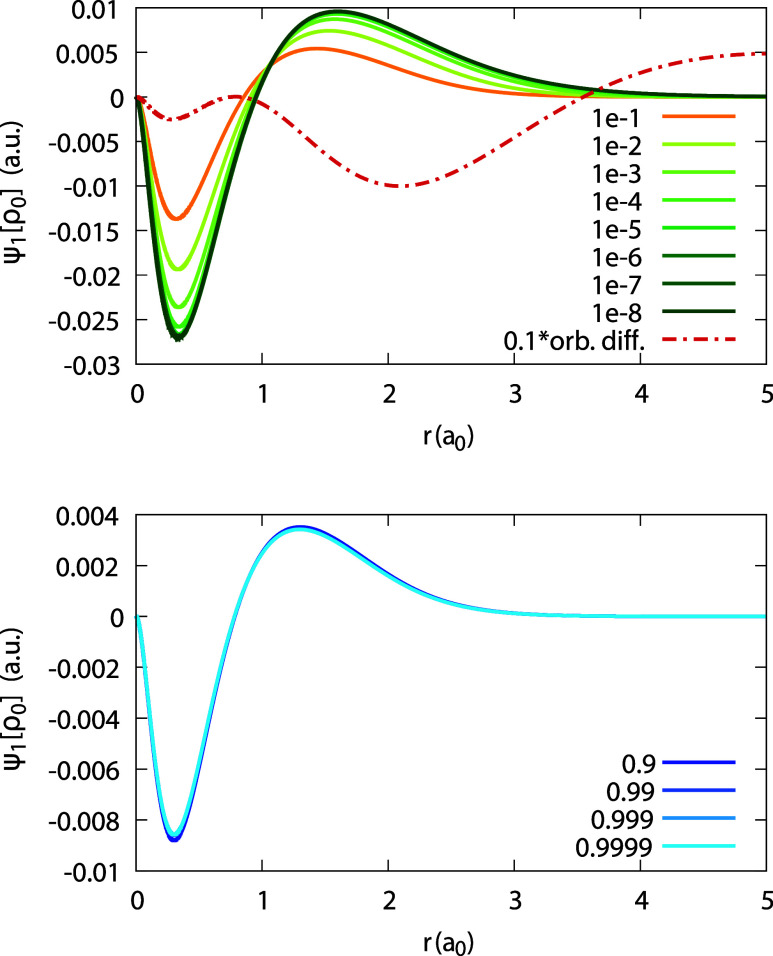
Quantity ψ_1_[ρ_0_](*r*;α) (defined in text), obtained for the Li atom, within the LDA, for different values of α. Top panel shows low α values, approaching 0^+^ to obtain _0_*c*_1_(*r*). Rhs of eq 31, divided by 10, is plotted on the top panel for comparison. Bottom panel features high α values, approaching 1^–^ to obtain _0_*d*_1_(*r*).

**Figure 19:**
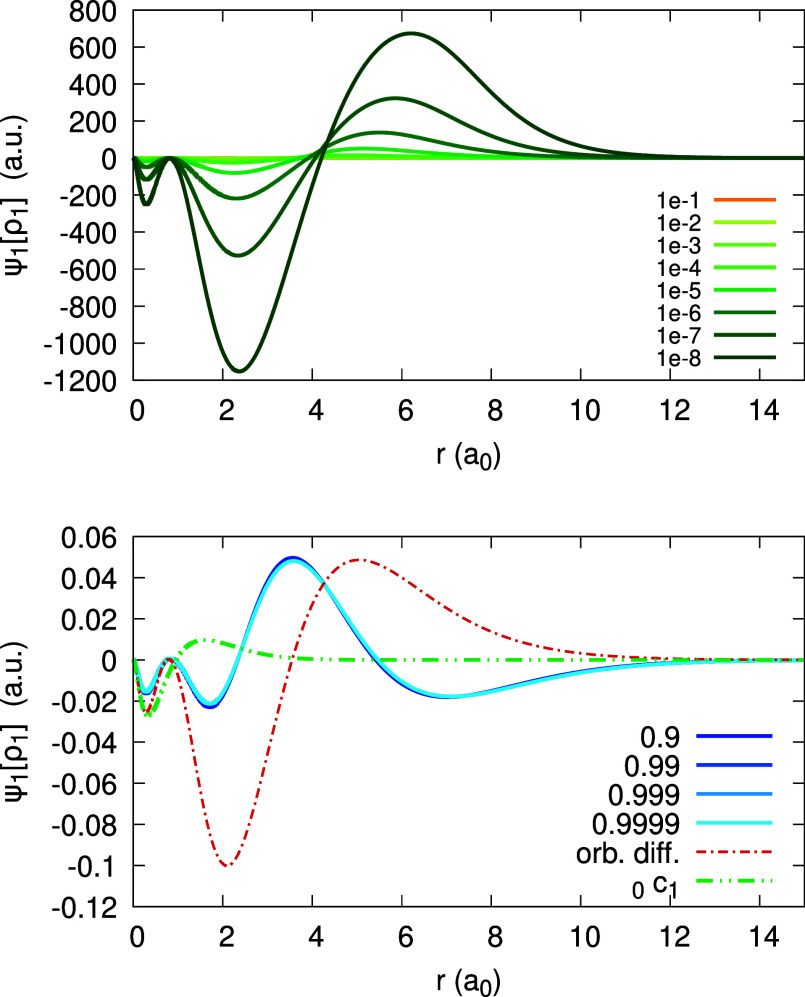
Quantity ψ_1_[ρ_1_](*r*;α) (defined in text), obtained for the Li atom, within the LDA, for different values of α. Top panel shows low α values, approaching 0^+^ in a failed attempt to obtain _1_*c*_1_(*r*). Bottom panel shows values that approach the limit 1^–^, to obtain _1_*d*_1_(*r*). Rhs of eq 31 and the coefficient _0_*c*_1_(*r*) are plotted for comparison.

The expansion coefficients _0_*c*_1_(**r**), _0_*d*_1_(**r**) and _1_*d*_1_ (**r**) can be obtained and are given in Figures 18 and 19 (bottom), as limits of ψ_1_[ρ_0_](**r**;α) and ψ_1_[ρ_1_](**r**;α). Due to the deviation of the density from piecewise-linearity, there is a large difference between _0_*c*_1_(**r**) and  (Figure 18(top)) and between each of them and _1_*d*_1_(**r**) (Figure 19(bottom)).

In contrast, the attempt to obtain the expansion coefficient _1_*c*_1_(**r**) for Li with LDA, as the limit lim_α→0^+^_ ψ_1_[ρ_1_](**r**; α) failed, as shown in Figure 19(top). As α decreases to 0^+^, the magnitude of ψ_1_[ρ_1_] becomes larger and larger, until it diverges. Since , and since the coefficients _0_*c*_1_(**r**) and _0_*d*_1_(**r**) are well-converged, we realize that the derivative of the HOMO,  diverges for Li. This statement has been explicitly verified numerically (cf. eqs 30 and 32).

It is emphasized that the HOMO itself smoothly changes as α → 0^+^; see Figure 20 in the SI, where the quantity
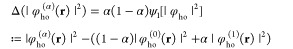
48is plotted. It is just that  does not decay fast enough with α, and then when ψ_1_[ρ_1_] (or ψ_1_[|φ_ho_|^2^]) is obtained via division by α(1 – α) (see eq 46), the result diverges. A similar situation with divergence of _1_*c*_1_(**r**) has also been observed for Li with invLDA and for Na and K (see the SI, Section V).

## 4 Discussion

**Figure 20:**
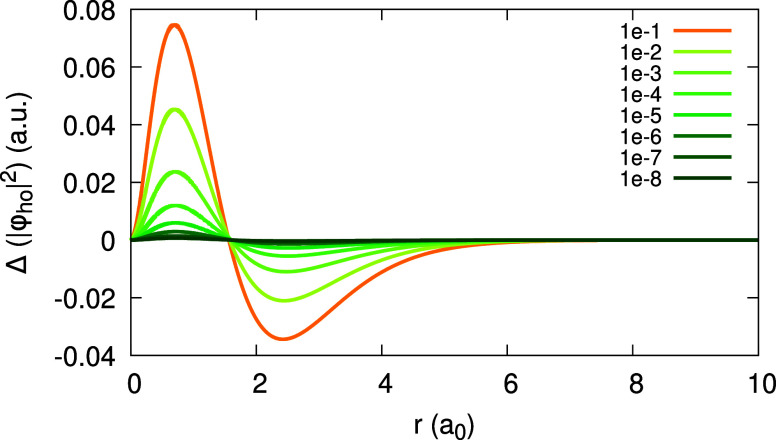
Quantity  for the H atom with the LDA, for various values of α (see Legend).

To deeper understand the differences between the anomalous situation with Li versus the standard Ag case, we first address arguably the simplest system of all–the H atom with α electrons (α ∈ [0, 1]). In this case, ρ_0_ = 0 and ρ_1_ equals the HOMO, |φ_1_(**r**)|^2^. Within LDA, H shows a behavior similar to that of Li: whereas *v*_1_(**r**) (= _1_*d*_1_(**r**) in this case) converges, *u*_1_(**r**) (= _1_*c*_1_(**r**)) diverges. The HOMO itself is smoothly varying with α, as Figure 20 shows. By direct observation, one can notice a scaling property of Δ(|φ_1_(**r**)|^2^): If one divides each curve in Figure 20 by a spatially constant, but α-dependent factor, such that the first peak of the resultant function always equals 1, all the scaled curves almost coincide (see Figure 18 of the SI). Therefore, the HOMO difference can be approximately described as Δ(|φ_1_(**r**)|^2^) ≈ *F*(α) *f*(**r**), where *f*(**r**) is the scaled spatial function (Figure 18 of the SI), and *F*(α) is obtained from the scaling analysis. Fitting the data for H on a log–log scale, we find that *F*(α) ≈ *F*_0_ α^γ^, where *F*_0_ ≈ 0.17 and γ ≈ 0.295 for H with LDA (Figure 19 of the SI). Therefore,  in our case, and since γ < 1, the aforementioned derivative diverges. Performing a similar analysis for Li results with LDA, yields a fit, which, although less accurate, yields *F*_0_ ≈ 0.079 and γ ≈ 0.476 (Figure 20 of the SI). The fact that the behavior of the first α-derivative of the HOMO at 0^+^ behaves as α^γ^ with γ < 1 is another (class of) deviation of approximate xc functionals from the expected exact behavior.

**Figure 21:**
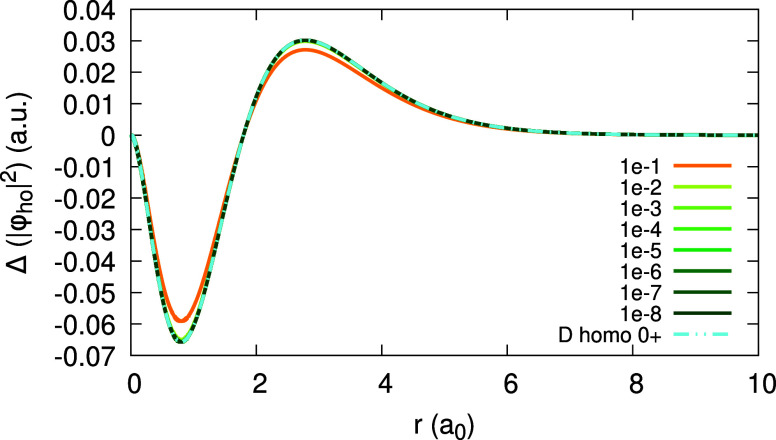
Quantity  for the H atom with invLDA, for various values of α (see Legend).

Finally, for H with invLDA, we find the following intriguing result. While *v*_1_(**r**) converges and *u*_1_(**r**) diverges, as before, the HOMO difference Δ(|φ_1_(**r**)|^2^) appears as in Figure 21: all the curves closely fit, no scaling is required. Consequently, *u*_1_(**r**) ∼ 1/α, which is fundamentally incorrect, but the more interesting point is that Δ(|φ_1_(**r**)|^2^) does not vanish as α → 0^+^, as it should. In other words, in H with invLDA there is a discontinuity in the HOMO orbital density: .

The reason for this new discontinuity can be clearly identified in the case of H: this is the self-interaction error of LDA. For H the density at fractional α equals , where  is the lowest squared orbital of H solved with LDA. It is different from the true 1s orbital of H due to self-interaction. Since invLDA enforces piecewise-linearity of the density for any fractional α, in H this means that the density must equal *n*(**r**) = α *n*_1_(**r**); for H the rhs here equals . Therefore, in invLDA the HOMO is forced to be , for all α ∈ (0, 1]. However,  is a different orbital: it is the lowest orbital of the “empty LDA H”, for which there is no self-interaction: the KS potential is simply the external attraction of the nucleus and  is then the true 1s orbital of H. For the exact functional (or at least, for functionals that are devoid of one-electron self-interaction) this discontinuity will not appear, but for many other approximations it is to be expected.

Investigation of the H atom, within the LDA and invLDA, may suggest an intuitive explanation of why the _1_*c*_1_(**r**) coefficient diverges in Li, Na and K. First, we note that all the atoms where we found a diverging _1_*c*_1_(**r**) belong to the alkali metals, where all the electrons reside in closed shells, except the HOMO, which we fractionally occupy by changing α. To strengthen this statement, calculations for Li-like ions, namely Be^+^, B^2+^, C^3+^, N^4+^ and Al^10+^, were performed, and in all these cases the _1_*c*_1_(**r**) coefficient is diverging (see the SI, Section V for details). This suggests that the scenario of divergence can be related to occupying a new electronic shell in an atom/ion, where the spatial overlap between the partially occupied HOMO and the closed-shell electrons is relatively low. In such a case, given that the relaxation of the closed-shell electrons is relatively weak (compare the magnitude of ψ_1_[ρ_0_] to that of ψ_1_[*n*] for Li vs Ag), the system can be approximately viewed as a H-like system, with the core electrons mainly screening the charge of the nucleus. Then, the burden of reproducing the piecewise-linearity of the density, exactly or approximately, lies mainly on the HOMO. This in turn may cause the HOMO  to equal , for most of the α-range, and, as in the case of H with invLDA, a discontinuity, or a very sharp change, in the HOMO orbital may build up as α → 0^+^. In terms of the 2pTE expansion, this means that the *u*_1_(**r**) and the _1_*c*_1_(**r**) coefficients diverge.

## 5 Conclusions and Outlook

In this contribution, I focused on the exact property of piecewise-linearity of the electron density and investigated how this property is reflected in the KS system. Removing the deviation of the electron density from the piecewise-linear regime is important for mitigating density-driven errors in DFT calculations. To this end, it is useful to know the exact and the approximate dependence of the total density and density ingredients on the electron number. The present contribution made a step in this direction, by investigating the α-dependence of the density, using the two-point Taylor expansion (2pTE). In particular, the total ensemble density *n*(**r**), the subdensities  and  and the HOMO orbital density  were addressed.

By employing the 2pTE method and expanding the aforementioned quantities in α around 0^+^ and 1^–^, it has been shown that the piecewise-linearity requirement creates a connection between the expansion coefficients _0_*c*_*k*_(**r**) of  and _1_*d*_*k*_(**r**) of  (eq 29). Furthermore, the coefficients _1_*c*_*k*_(**r**) and _0_*d*_*k*_(**r**) were related to the α-derivatives of the HOMO orbital density. Therefore, if one obtains exact or approximate expressions for the α-dependence of the HOMO, the Taylor coefficients _*p*_*c*_*k*_(**r**) and _*p*_*d*_*k*_(**r**) can be fully determined. However, the piecewise-linearity requirement itself does not constrain the HOMO’s α-behavior. For this reason, four different regimes for the HOMO were considered in detail, including the well-known frozen regime and the linear regime.

Numerically, the 2pTE coefficients have been extracted for selected atomic systems. The numerical effort required to obtain accurate, converged coefficients proved to be significant even in relatively simple cases. Usage of the invLDA and invPBE functionals showed that enforcement of piecewise-linearity significantly improves satisfying of exact properties, particularly eq 31. Furthermore, it has been shown that even in simple, atomic systems, with common functionals (LDA and PBE), the HOMO is far from being frozen; even the linear approximation may not be accurate enough. In addition, enforcing piecewise-linearity can result even in a more complex dependence of the HOMO on α, as we saw above for Ag.

Moreover, not in all systems the subdensity ρ_1_ (and subsequently the HOMO) is 2pTE-expandable. For H, Li, Na, K and Li-like ions, the coefficient _1_*c*_1_ diverges as α^γ–1^, with γ < 1. This circumstance limits the applicability of the 2pTE approach. Whether the aforementioned divergence is an artifact of some xc approximations in certain atoms or a real feature of many-electron systems, remains a question of future research. Here, however, we showed that in the case of H this feature is directly related to one-electron self-interaction; an attempt to enforce piecewise-linearity in this case may come at the price of a discontinuity in the HOMO orbital density. Relying on the H case, an intuitive explanation of why divergence of the _1_*c*_1_ coefficient occurs in Group 1 atoms and ions has been suggested, relating the aforementioned divergence to the low overlap between the partially occupied HOMO and the core electrons. This explanation suggests that diverging 2pTE coefficients can be expected also in molecular systems, if the overlap between the HOMO and other orbitals is low. In the context of molecular systems, such a scenario may occur in cases of charge transfer and in certain dissociation scenarios.

This work focused on finite systems. However, questions of piecewise linearity and derivative discontinuity are of great importance also in infinite periodic solids. There, however, it becomes impossible to directly address the system’s cation or anion and monitor how the system changes when varying the electron number. Because such systems are normally represented by a unit cell with periodic boundary conditions, varying the electron number per unit cell means adding or subtracting charge from each replica of the unit cell and therefore an infinitely large charge from the system as a whole. Introducing a compensating uniform background, thus keeping the overall system neutral, may solve the problem, but this hinders the straightforward use of total energy differences and of density differences. Nonetheless, addressing solids with methods described in this work, e.g., by means of considering a series of finite, but increasingly large systems that approach a crystalline solid as a limit, is a promising direction of future research.

Further research directions include, first, the 2pTE expansion of other quantities, including the KS potential and the corresponding orbitals, as well as quantities beyond DFT, such as the 1-body reduced density matrix, the interacting and the KS Green functions, the corresponding self-energy and more. Second, cases such as Li should receive a different formal treatment. Third, investigation of other chemical and physical processes, where many-electron subsystems have a varying electron number, with methods similar to those developed here can prove useful. Such processes include dissociation, excitation, charge transfer and adsorption–all extremely relevant in chemistry and materials research.

## Supporting Information Available

Additional technical data on numerical convergence of the Ag atom and the scaling analysis for the H and Li atoms and additional numerical data for the B, Al, Cu, and Au atoms, mentioned in the main text, but not discussed in detail, due to the qualitative similarity of their results to the Ag atom (typical case) and data on the Na and K atoms, as well as the Li-like ions Be^+^, B^2+^, C^3+^, N^4+^, and Al^10+^ (atypical cases) (PDF)
