# Supplementary material for: How the Piecewise-Linearity Requirement for the Density Affects Quantities in the Kohn–Sham System
Source: J Chem Theory Comput. 2024 Dec 16;21(1):155–69. doi: 10.1021/acs.jctc.4c01152 (PMC11736690; doi:10.1021/acs.jctc.4c01152)
Supplement: Supplementary file 1 — ct4c01152_si_001.pdf [file ct4c01152_si_001.pdf]

Supporting Information to the article: “How the piecewise-linearity requirement for the density affects quantities in the Kohn-Sham system”

Eli Kraisler*

Fritz Haber Center for Molecular Dynamics and Institute of Chemistry, The Hebrew University of Jerusalem, 9190401 Jerusalem, Israel

(Dated: November 12, 2024)

This document presents supporting information, in a graphical form, for the atomic systems mentioned in the main text, but not discussed in detail due to their similarity to the results for Ag (typical cases) or to Li (atypical cases). Furthermore, results for Ag, Li and H, which are described in the main text in words, but not presented graphically, are presented graphically here.

I. The function $\psi_1[n](\mathbf{r};\alpha)$ for Ag with invLDA and invPBE

In the main text, the quantity $\psi_1[n]$ has been defined in Eq. (46) and presented graphically for Ag. The limits of this quantity at $\alpha \to 0^+$ and $\alpha \to 1^-$ yield the expansion coefficients $\tilde{c}_1(\mathbf{r})$ and $\tilde{d}_1(\mathbf{r})$, respectively.

For the exact xc functional, the density is piecewise-linear and therefore the quantity $\psi_1[n](\mathbf{r};\alpha)$ is zero, for all values of α and $\mathbf{r}$. For approximate xc functionals, such as the LDA and the PBE, where piecewise-linearity of the density is not closely obeyed, the function $\psi_1[n]$ significantly varies from zero. This has been shown in Fig. 1 of the main text.

In contrast, for invLDA and invPBE, where piecewise-linearity is enforced, $\psi_1[n](\mathbf{r};\alpha) = 0$. The deviation of this quantity from zero is due to the finite numerical accuracy is the inversion procedure. Fig. 1 shows $\psi_1[n](\mathbf{r};\alpha)$ for invLDA and invPBE. We can clearly see that the requirement that the magnitude of $\psi_1[n]$ stays below 0.0001 is satisfied.

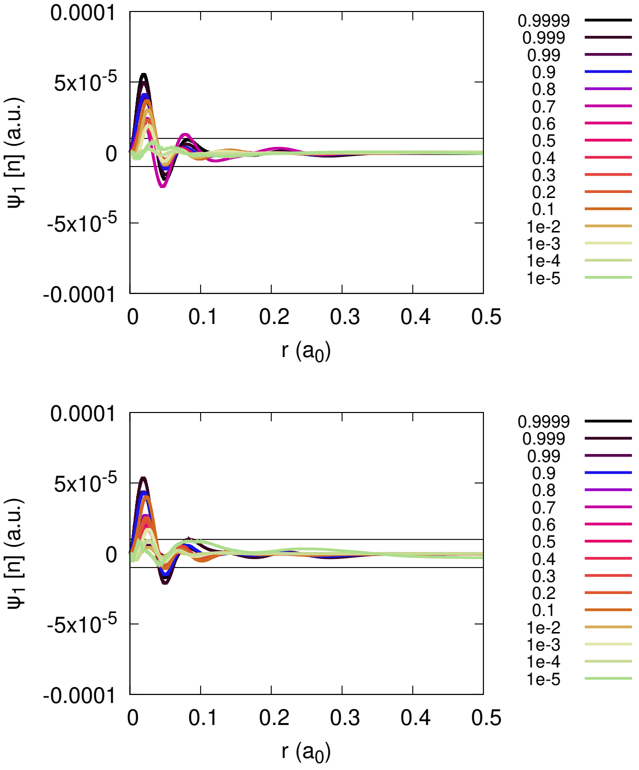

FIG. 1. The quantity $\psi_1[n](r;\alpha)$ (defined in the main text), obtained for the Ag atom, within the invLDA (top) and invPBE (bottom), for different values of α (see Legend)

II. Typical cases: B, Al, Cu and Au

Section III.B of the main text presented the Ag atom as the typical case. Here we present results also for the atoms B, Al, Cu and Au. For each system, we present the quantities $\psi_1[\rho_0]$ and $\psi_1[\rho_1]$, for each xc approximation. The coefficients ${}_0c_1(\mathbf{r})$, ${}_0d_1(\mathbf{r})$, ${}_1c_1(\mathbf{r})$, ${}_1d_1(\mathbf{r})$, as well as $u_1(\mathbf{r})$ and $v_1(\mathbf{r})$, can be deduced from the presented data, considering the limits $\alpha \to 0^+$ and $\alpha \to 1^-$.

* eli.kraisler@mail.huji.ac.il

A. The B atom

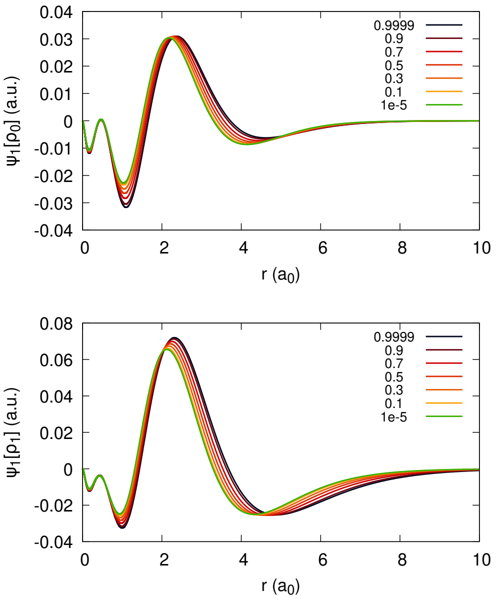

FIG. 2. The quantities $\psi_1[\rho_0](r;\alpha)$ (top) and $\psi_1[\rho_1](r;\alpha)$ (bottom), defined in the main text, obtained for the B atom, within the LDA, for different values of α (see Legend)

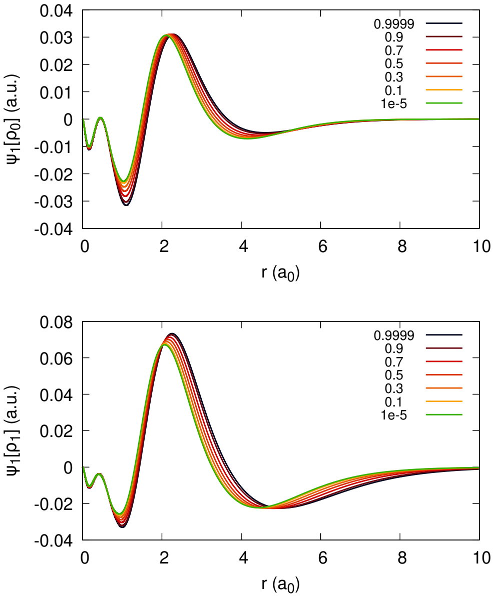

FIG. 3. The quantities $\psi_1[\rho_0](r;\alpha)$ (top) and $\psi_1[\rho_1](r;\alpha)$ (bottom), defined in the main text, obtained for the B atom, within the PBE, for different values of α (see Legend)

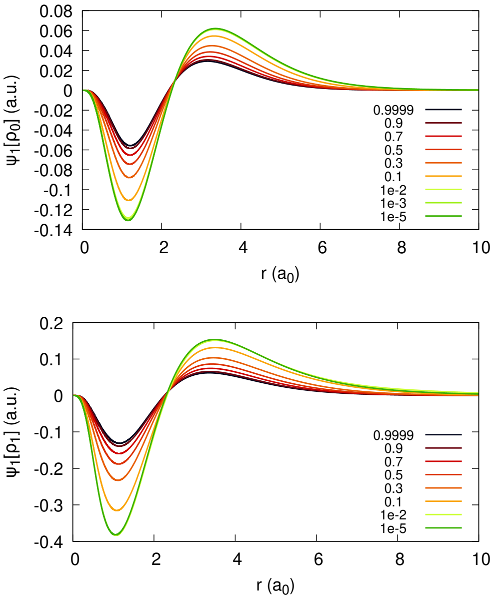

FIG. 4. The quantities $\psi_1[\rho_0](r;\alpha)$ (top) and $\psi_1[\rho_1](r;\alpha)$ (bottom), defined in the main text, obtained for the B atom, within the invLDA, for different values of α (see Legend)

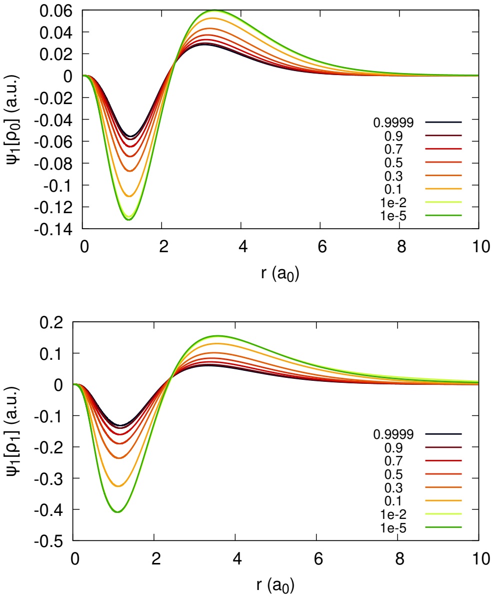

FIG. 5. The quantities $\psi_1[\rho_0](r;\alpha)$ (top) and $\psi_1[\rho_1](r;\alpha)$ (bottom), defined in the main text, obtained for the B atom, within the invPBE, for different values of α (see Legend)

B. The Al atom

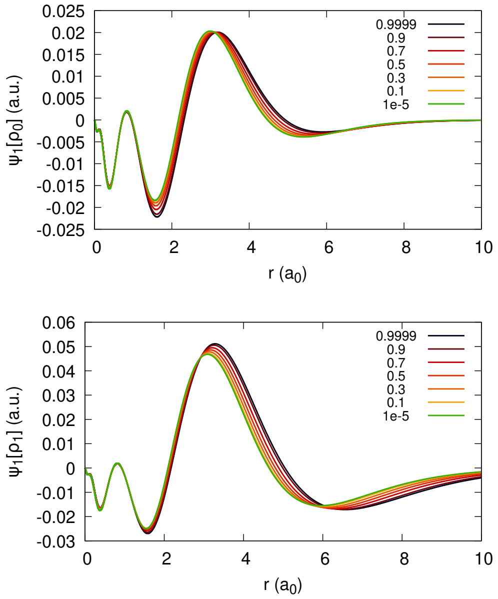

FIG. 6. The quantities $\psi_1[\rho_0](r;\alpha)$ (top) and $\psi_1[\rho_1](r;\alpha)$ (bottom), defined in the main text, obtained for the Al atom, within the LDA, for different values of α (see Legend)

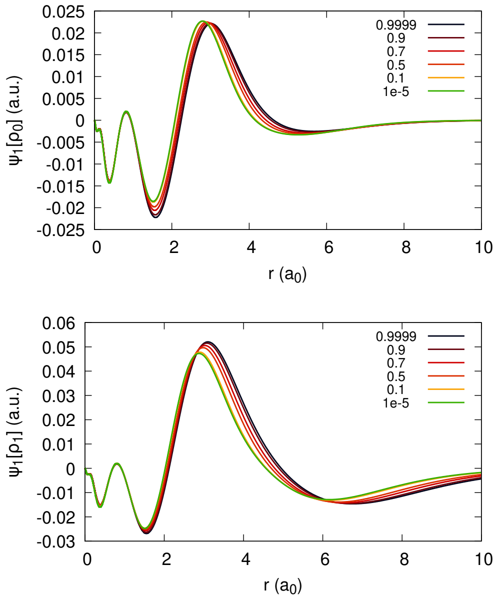

FIG. 7. The quantities $\psi_1[\rho_0](r;\alpha)$ (top) and $\psi_1[\rho_1](r;\alpha)$ (bottom), defined in the main text, obtained for the Al atom, within the PBE, for different values of α (see Legend)

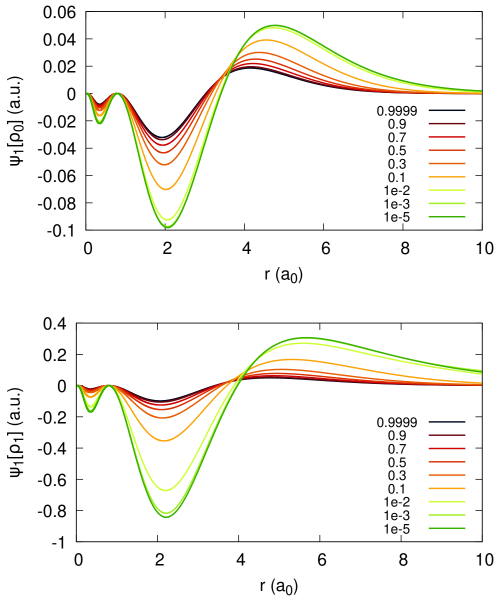

FIG. 8. The quantities $\psi_1[\rho_0](r;\alpha)$ (top) and $\psi_1[\rho_1](r;\alpha)$ (bottom), defined in the main text, obtained for the Al atom, within the invLDA, for different values of α (see Legend)

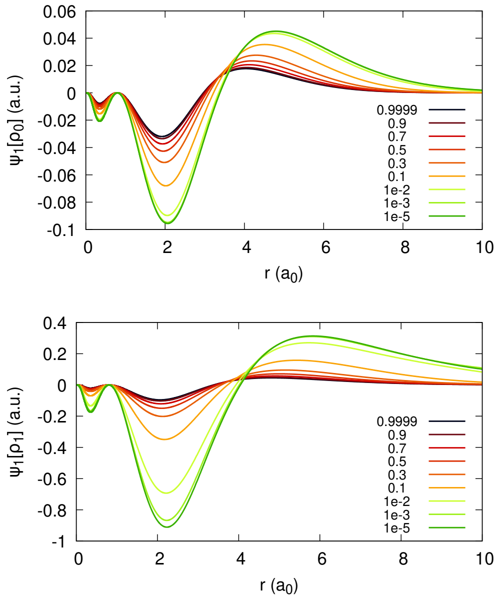

FIG. 9. The quantities $\psi_1[\rho_0](r;\alpha)$ (top) and $\psi_1[\rho_1](r;\alpha)$ (bottom), defined in the main text, obtained for the Al atom, within the invPBE, for different values of α (see Legend)

C. The Cu atom

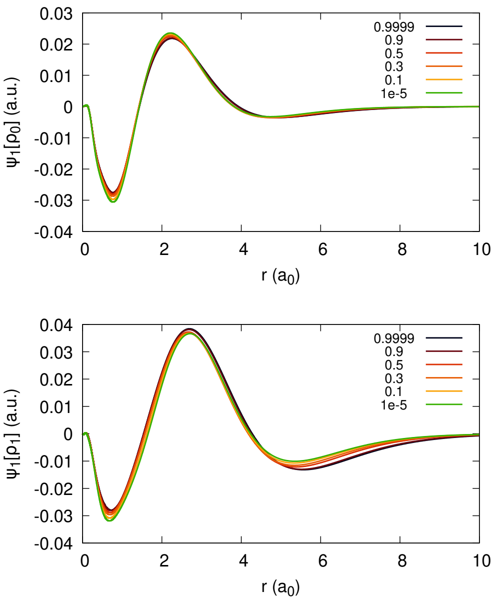

FIG. 10. The quantities $\psi_1[\rho_0](r;\alpha)$ (top) and $\psi_1[\rho_1](r;\alpha)$ (bottom), defined in the main text, obtained for the Cu atom, within the LDA, for different values of α (see Legend)

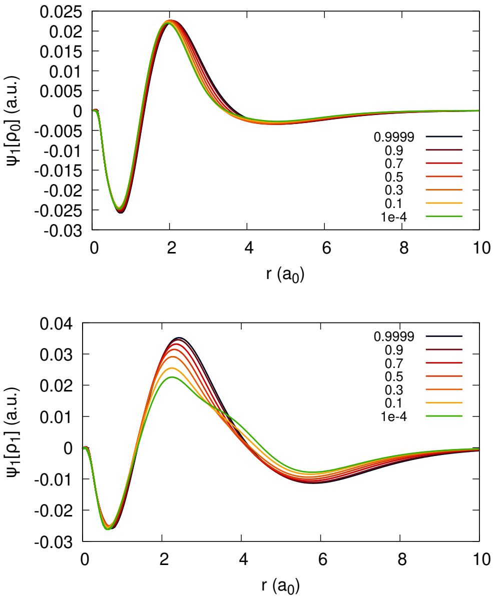

FIG. 11. The quantities $\psi_1[\rho_0](r;\alpha)$ (top) and $\psi_1[\rho_1](r;\alpha)$ (bottom), defined in the main text, obtained for the Cu atom, within the PBE, for different values of α (see Legend)

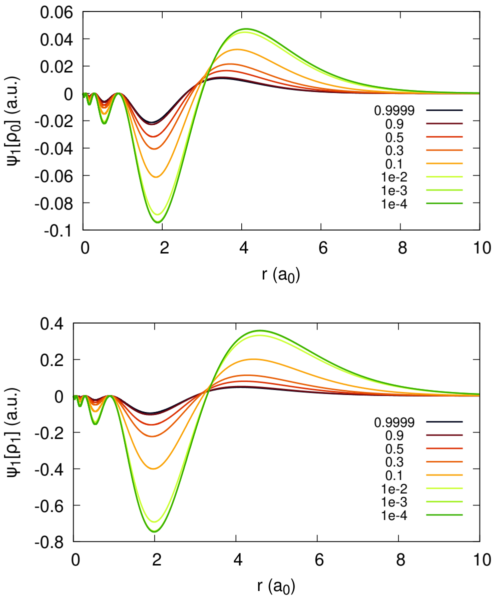

FIG. 12. The quantities $\psi_1[\rho_0](r;\alpha)$ (top) and $\psi_1[\rho_1](r;\alpha)$ (bottom), defined in the main text, obtained for the Cu atom, within the invLDA, for different values of α (see Legend)

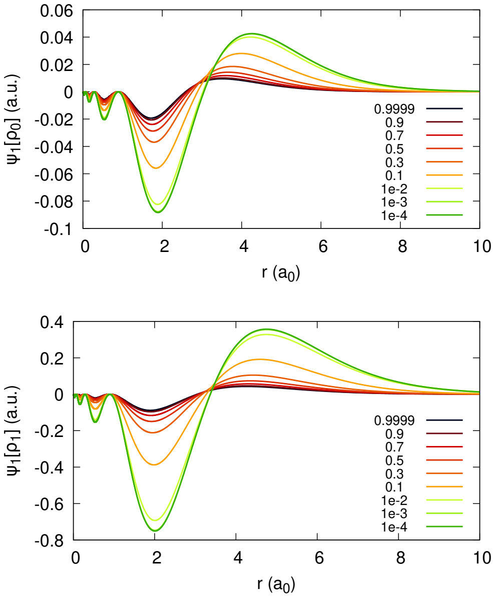

FIG. 13. The quantities $\psi_1[\rho_0](r;\alpha)$ (top) and $\psi_1[\rho_1](r;\alpha)$ (bottom), defined in the main text, obtained for the Cu atom, within the invPBE, for different values of α (see Legend)

D. The Au atom

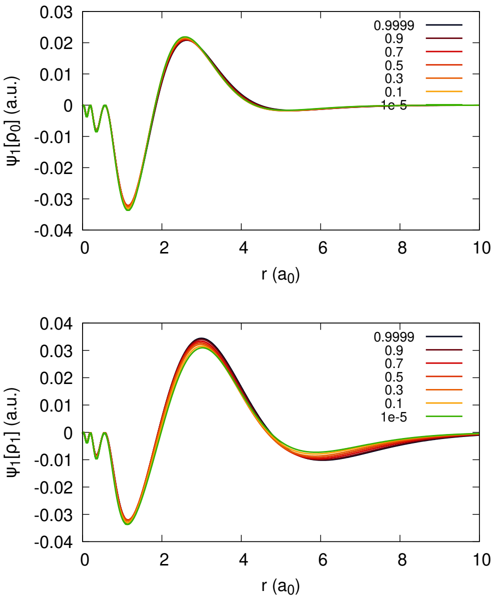

FIG. 14. The quantities $\psi_1[\rho_0](r;\alpha)$ (top) and $\psi_1[\rho_1](r;\alpha)$ (bottom), defined in the main text, obtained for the Au atom, within the LDA, for different values of α (see Legend)

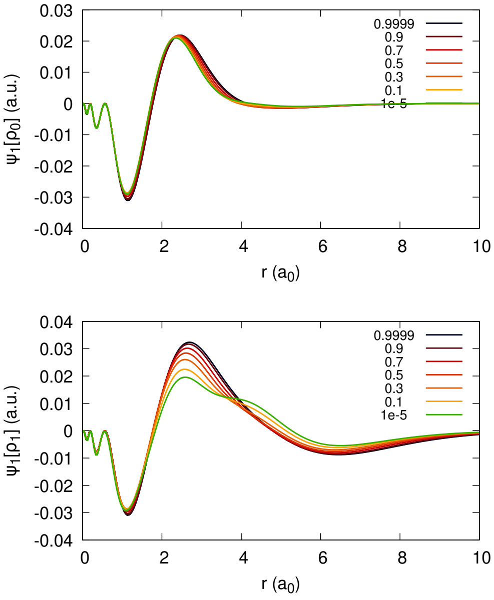

FIG. 15. The quantities $\psi_1[\rho_0](r;\alpha)$ (top) and $\psi_1[\rho_1](r;\alpha)$ (bottom), defined in the main text, obtained for the Au atom, within the PBE, for different values of α (see Legend)

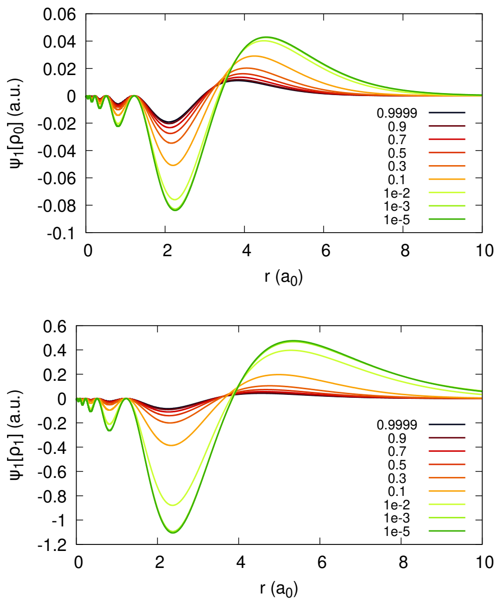

FIG. 16. The quantities $\psi_1[\rho_0](r;\alpha)$ (top) and $\psi_1[\rho_1](r;\alpha)$ (bottom), defined in the main text, obtained for the Au atom, within the invLDA, for different values of α (see Legend)

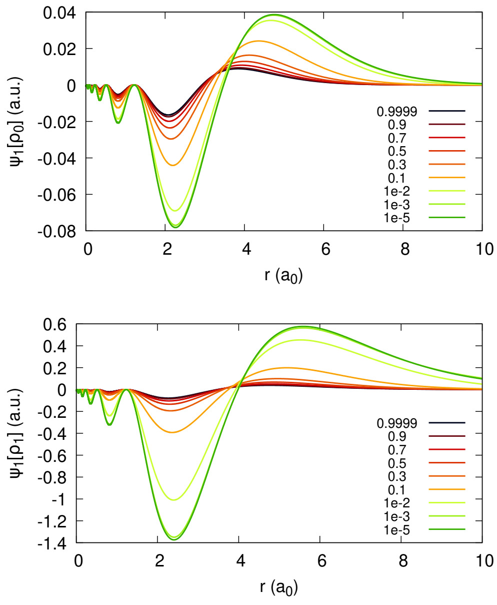

FIG. 17. The quantities $\psi_1[\rho_0](r;\alpha)$ (top) and $\psi_1[\rho_1](r;\alpha)$ (bottom), defined in the main text, obtained for the Au atom, within the invPBE, for different values of α (see Legend)

III. The H atom

Figure 20 (top) shows the quantity

$$\Delta\left(|\varphi_{\rm ho}^{(\alpha)}(\mathbf{r})|^2\right) = \alpha(1-\alpha)\psi_1[|\varphi_{\rm ho}|^2] \\ := |\varphi_{\rm ho}^{(\alpha)}(\mathbf{r})|^2 - \left((1-\alpha)|\varphi_{\rm ho}^{(0)}(\mathbf{r})|^2 + \alpha|\varphi_{\rm ho}^{(1)}(\mathbf{r})|^2\right) \quad (1)$$

for H with LDA (identical to Fig. 20 of the main text). As $\alpha \to 0^+$, this quantity vanishes, as expected. However, it does not vanish quickly enough with α, which causes the divergence in the coefficients $u_1(\mathbf{r})$ $(={}_1c_1(\mathbf{r})$ for H). Figure 20 (bottom) shows the scaled quantity $\Delta(|\varphi_{\rm ho}^{(\alpha)}(\mathbf{r})|^2)$, which is obtained from the original data by dividing each curve by a different constant, such that its first peak equals 1, for all α. Remarkably, all the scaled curves closely overlap. Therefore, the representation $\Delta(|\varphi_{\rm ho}^{(\alpha)}(\mathbf{r})|^2) = F(\alpha)f(\mathbf{r})$ is accurate. The scaled curve $f(\mathbf{r})$ in nothing else but Figure 20 (bottom), whereas $F(\alpha)$ is presented in Fig. 19. A linear fit (on the log-log plot) yields the following approximate expression: $F(\alpha) \approx F_0\alpha^\gamma$, where $F_0 \approx 0.17$ and $\gamma \approx 0.295$

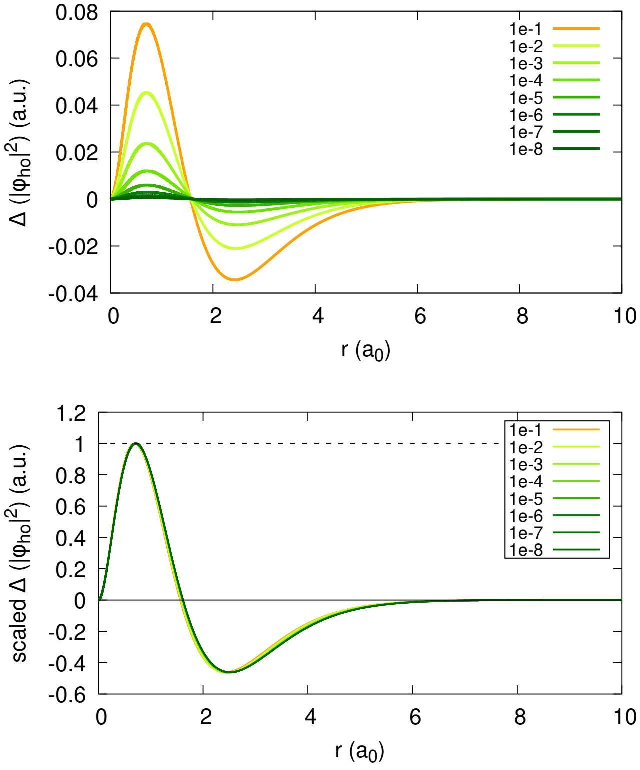

FIG. 18. (top) The quantity $\Delta(|\varphi_{\rm ho}^{(\alpha)}(\mathbf{r})|^2)$ for the H atom with the LDA, for various values of α (see Legend). (bottom) The same quantity, scaled such that its first peak equals 1, for all α.

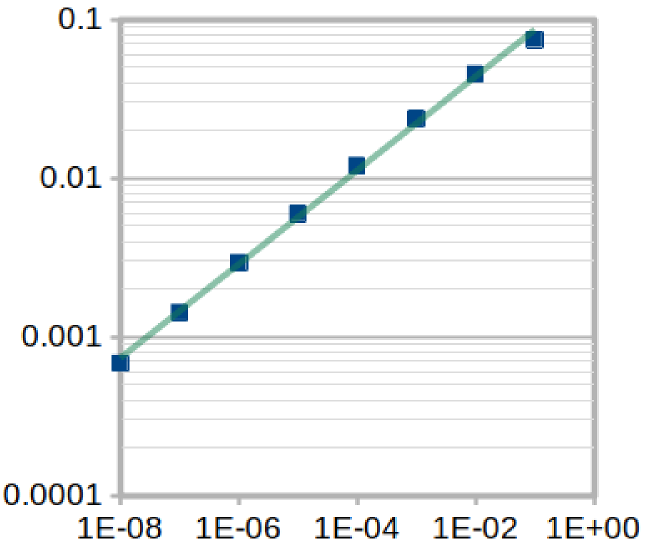

FIG. 19. The scaling function $F(\alpha)$ (defined in text), obtained for the H atom with LDA, analysing $\Delta\left(|\varphi_{\rm ho}^{(\alpha)}(\mathbf{r})|^2\right)$ for $\alpha = 10^{-n}$, with $n = 1, 2, \cdots, 8$ (squares). Both the horizontal axis (α) and the vertical axis ($F(\alpha)$) are unitless logarithmic axes. The solid line is a linear fit (on a log-log plot).

IV. The Li atom

In Fig. 20 (top) the quantity $\Delta\left(|\varphi_{\rm ho}^{(\alpha)}(\mathbf{r})|^2\right)$ is plotted (see Eq. (1) for a definition). As $\alpha \to 0^+$, this quantity vanishes, but not fast enough with α, which causes the divergence in the coefficients $u_1(\mathbf{r})$ and ${}_1c_1(\mathbf{r})$ for Li (Fig. 19 (top) of the main text). In Fig. 20 (bottom) we see the scaled quantity $\Delta(|\varphi_{\rm ho}^{(\alpha)}(\mathbf{r})|^2)$, such that its outermost peak equals 1, for all α. For Li the scaling is less justified, because he scaled curves do not really overlap. Nonetheless, analysis of the amplitudes shows that $F(\alpha) \approx 0.079\alpha^{0.476}$.

In the following, we present in detail results for $\psi_1[\rho_0](r;\alpha)$ and $\psi_1[\rho_1](r;\alpha)$, in the limits $\alpha \to 0^+$ and $\alpha \to 1^-$, obtained for Li with invLDA, where piecewise-linearity of the density is assured. We find that ${}_1c_1$ diverges (Fig. 22). The coefficients ${}_0d_1$ and ${}_1d_1$ clearly converge and are presented in Fig. 21 (bottom) and 22 (bottom), respectively. Close correspondence between ${}_1d_1$ and $|\varphi_{N_0+1}^{(1)}(\mathbf{r})|^2 - |\varphi_{N_0+1}^{(0)}(\mathbf{r})|^2$ is observed also here. ${}_0c_1$ (Fig. 21 (top)) approaches its limit so slowly that values of $\alpha < 10^{-9}$ are required to obtain full convergence. This poses a numerical challenge, but it is clear that ${}_0c_1$ is not going to diverge, but reach $|\varphi_{N_0+1}^{(1)}(\mathbf{r})|^2 - |\varphi_{N_0+1}^{(0)}(\mathbf{r})|^2$ (shown there for comparison).

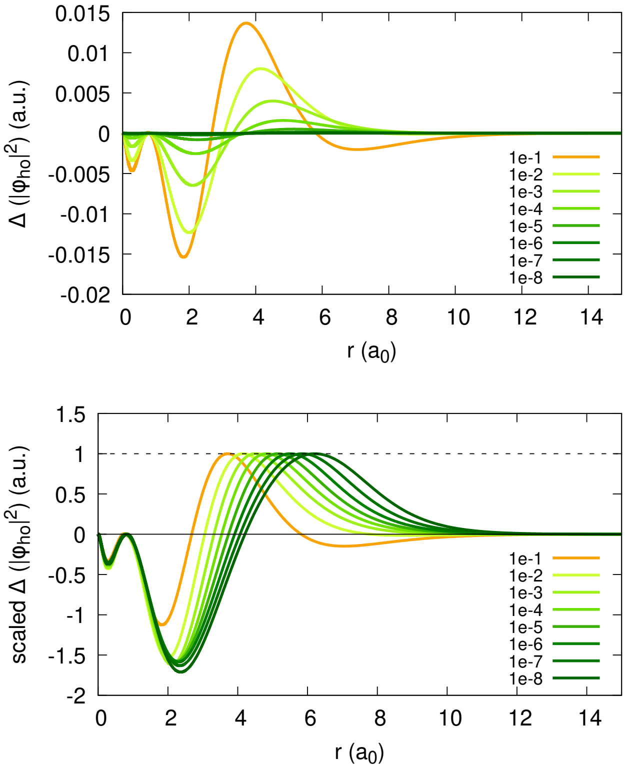

FIG. 20. (top) The quantity $\Delta(|\varphi_{\rm ho}^{(\alpha)}(\mathbf{r})|^2)$ for the Li atom with the LDA, for various values of α (see Legend). (bottom) The same quantity, scaled such that its fourth (outermost) peak equals 1, for all α.

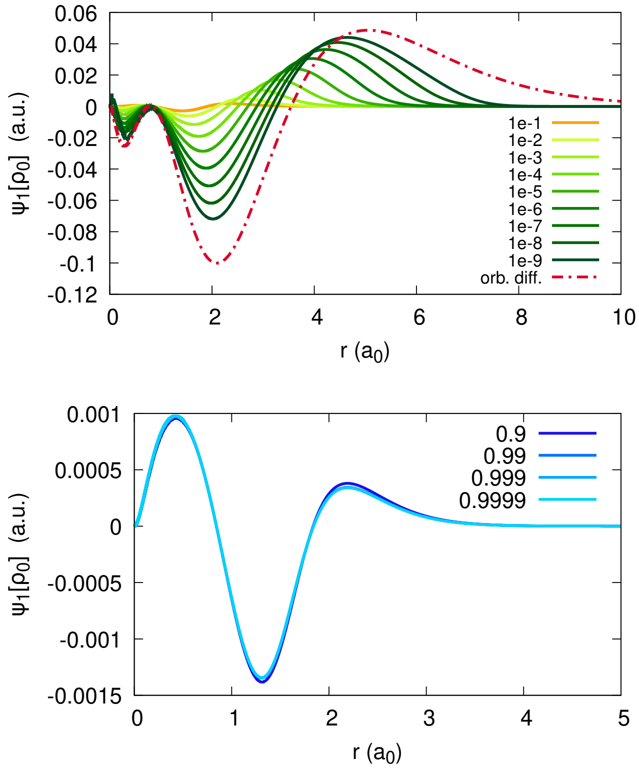

FIG. 21. The quantity $\psi_1[\rho_0](r;\alpha)$, obtained for the Li atom, within invLDA, for different values of α. The top panel shows low α values, approaching 0^+ to obtain ${}_0c_1(r)$. The rhs of Eq. (31) of the main text is plotted on the top panel for comparison. The bottom panel features high α values, approaching 1^- to obtain ${}_0d_1(r)$.

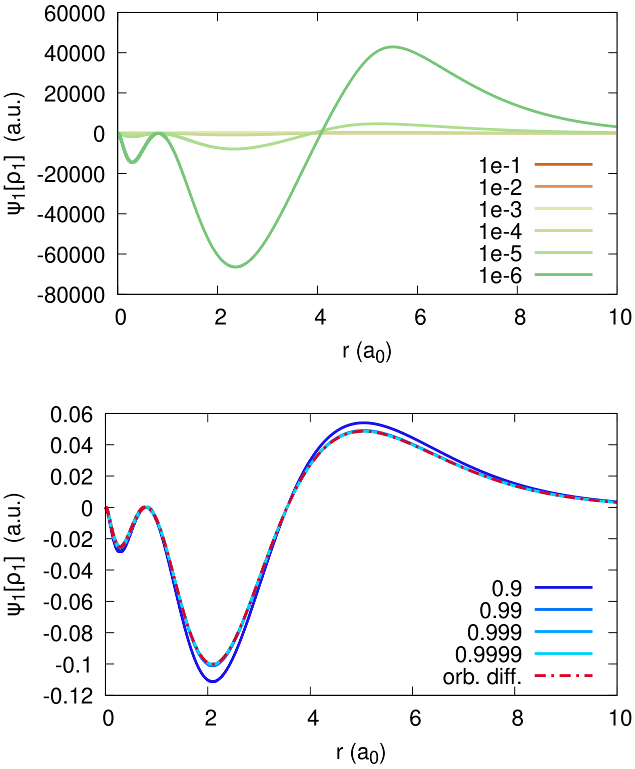

FIG. 22. The quantity $\psi_1[\rho_1](r;\alpha)$, obtained for the Li atom, within invLDA, for different values of α. The top panel shows low α values, approaching 0^+ to obtain ${}_1c_1(r)$. No convergence occurs. The bottom panel features high α values, approaching 1^- to obtain ${}_1d_1(r)$. The rhs of Eq. (31) of the main text is plotted on the top panel for comparison.

V. Atypical cases: Na, K, Be^+, B^{2+}, C^{3+}, N^{4+} and Al^{10+}

Section III.C in the main text presented the Li atom as the atypical case for the 2pTE, and Sec. IV presented also H. Here we present results for the atoms Na and K and the Li-like ions Be^+, B^{2+}, C^{3+}, N^{4+} and Al^{10+}. For each system, we present the quantities $\psi_1[\rho_0]$ and $\psi_1[\rho_1]$, numerically emphasizing the limits $\alpha \to 0^+$ and $\alpha \to 1^-$. From the following plots, it is apparent when the numerical limit can be reached (and therefore the corresponding coefficient function ${}_0c_1(\mathbf{r})$, ${}_0d_1(\mathbf{r})$, ${}_1c_1(\mathbf{r})$ or ${}_1d_1(\mathbf{r})$ can be obtained), and when the procedure diverges. Relying on the experience the systems presented in the main text, here we focus solely on the LDA.

A. The Na atom

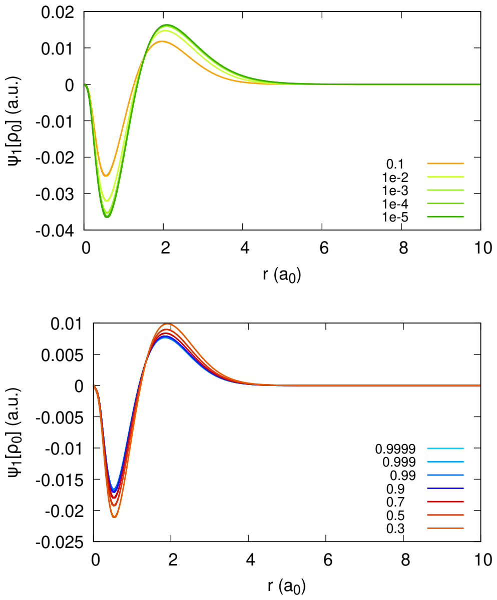

FIG. 23. The quantity $\psi_1[\rho_0](r;\alpha)$ in the limit $\alpha \to 0^+$ (top) and $\alpha \to 1^-$ (bottom) obtained for the Na atom, within the LDA

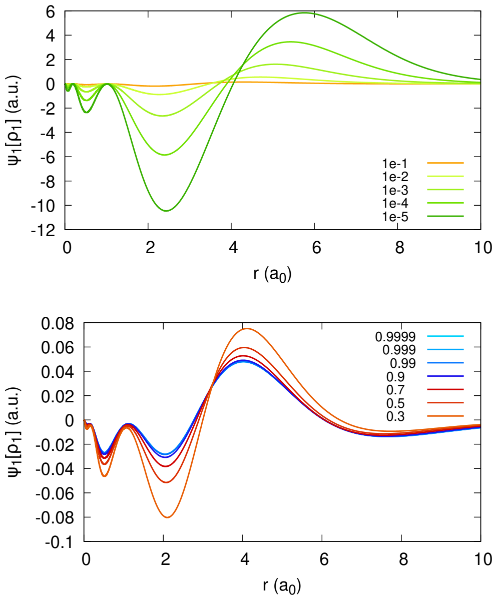

FIG. 24. The quantity $\psi_1[\rho_1](r;\alpha)$ in the limit $\alpha \to 0^+$ (top; diverges!) and $\alpha \to 1^-$ (bottom) obtained for the Na atom, within the LDA

B. The K atom

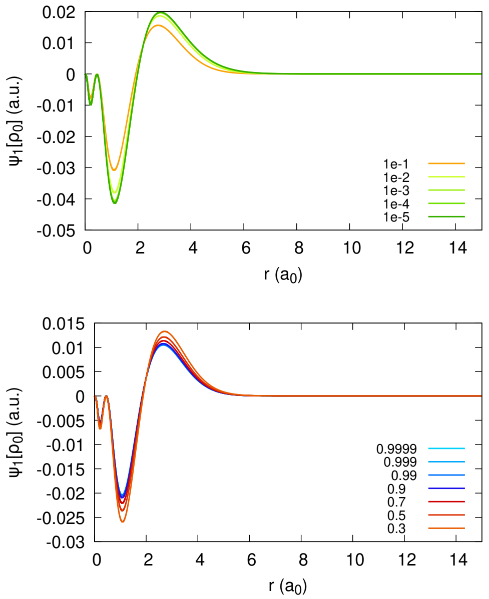

FIG. 25. The quantity $\psi_1[\rho_0](r;\alpha)$ in the limit $\alpha \to 0^+$ (top) and $\alpha \to 1^-$ (bottom) obtained for the K atom, within the LDA

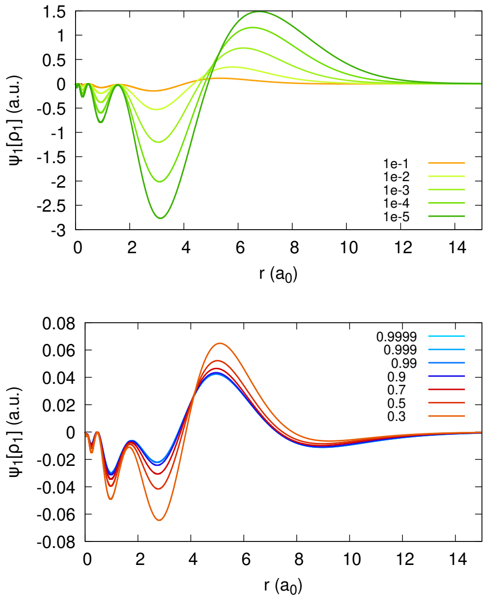

FIG. 26. The quantity $\psi_1[\rho_1](r;\alpha)$ in the limit $\alpha \to 0^+$ (top) and $\alpha \to 1^-$ (bottom) obtained for the K atom, within the LDA

C. The Be^+ ion

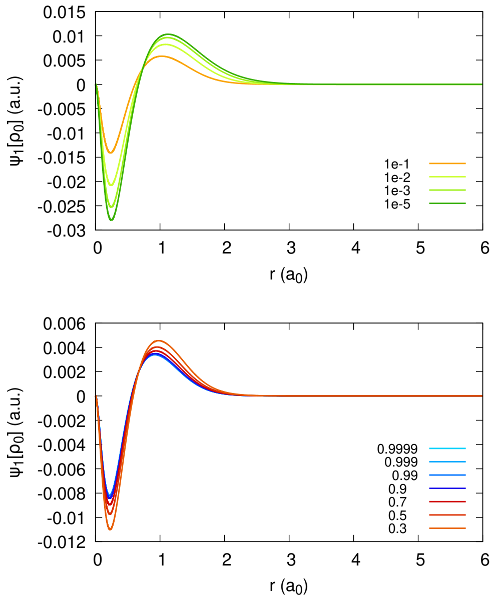

FIG. 27. The quantity $\psi_1[\rho_0](r;\alpha)$ in the limit $\alpha \to 0^+$ (top) and $\alpha \to 1^-$ (bottom) obtained for the Be^+ ion, within the LDA

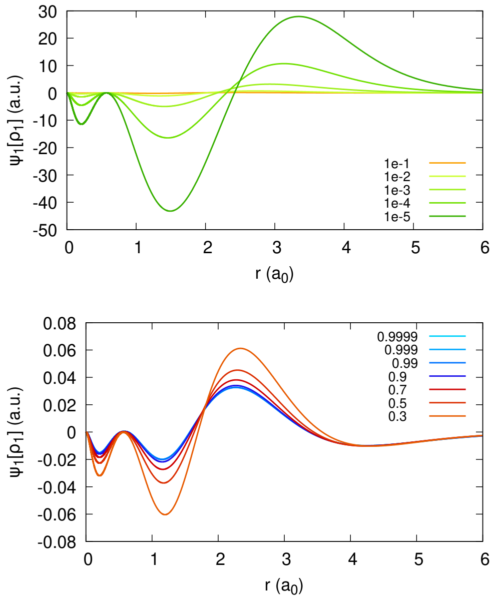

FIG. 28. The quantity $\psi_1[\rho_1](r;\alpha)$ in the limit $\alpha \to 0^+$ (top) and $\alpha \to 1^-$ (bottom) obtained for the Be^+ ion, within the LDA

D. The B^{2+} ion

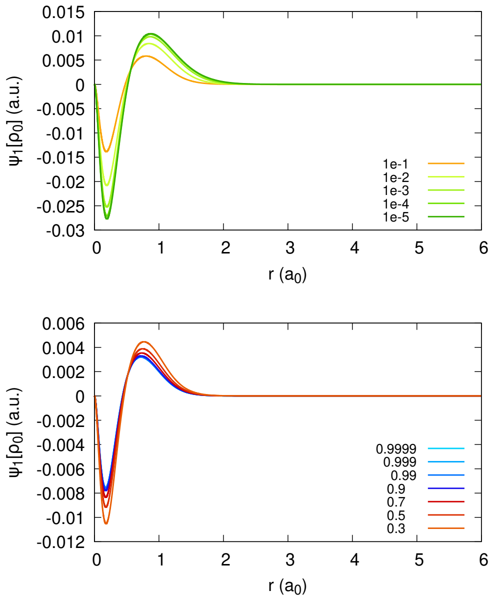

FIG. 29. The quantity $\psi_1[\rho_0](r;\alpha)$ in the limit $\alpha \to 0^+$ (top) and $\alpha \to 1^-$ (bottom) obtained for the B^{2+} ion, within the LDA

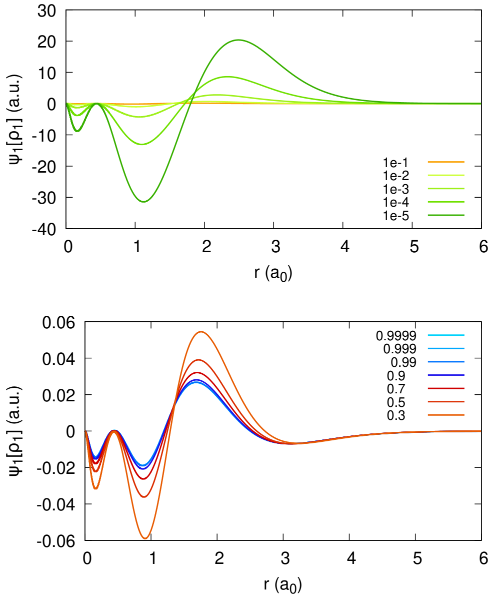

FIG. 30. The quantity $\psi_1[\rho_1](r;\alpha)$ in the limit $\alpha \to 0^+$ (top) and $\alpha \to 1^-$ (bottom) obtained for the B^{2+} ion, within the LDA

E. The C^{3+} ion

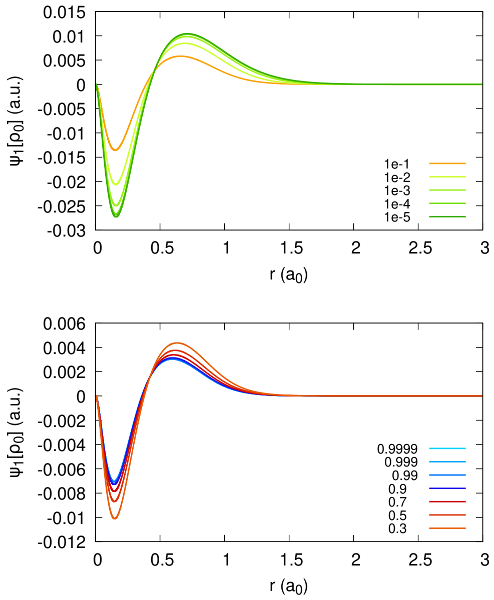

FIG. 31. The quantity $\psi_1[\rho_0](r;\alpha)$ in the limit $\alpha \to 0^+$ (top) and $\alpha \to 1^-$ (bottom) obtained for the C^{3+} ion, within the LDA

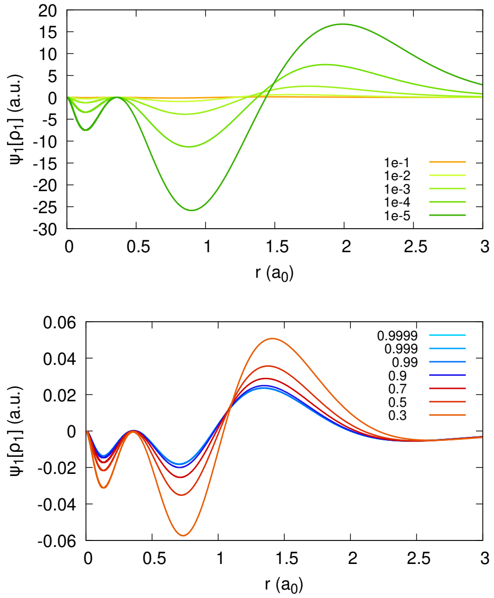

FIG. 32. The quantity $\psi_1[\rho_1](r;\alpha)$ in the limit $\alpha \to 0^+$ (top) and $\alpha \to 1^-$ (bottom) obtained for the C^{3+} ion, within the LDA

F. The N^{4+} ion

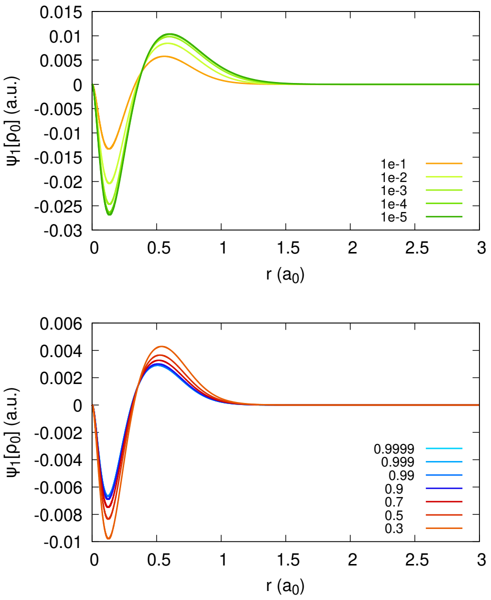

FIG. 33. The quantity $\psi_1[\rho_0](r;\alpha)$ in the limit $\alpha \to 0^+$ (top) and $\alpha \to 1^-$ (bottom) obtained for the N^{4+} ion, within the LDA

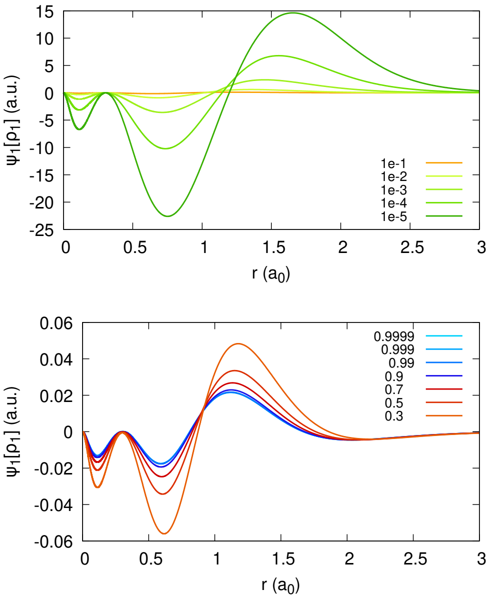

FIG. 34. The quantity $\psi_1[\rho_1](r;\alpha)$ in the limit $\alpha \to 0^+$ (top) and $\alpha \to 1^-$ (bottom) obtained for the N^{4+} ion, within the LDA

G. The Al^{10+} ion

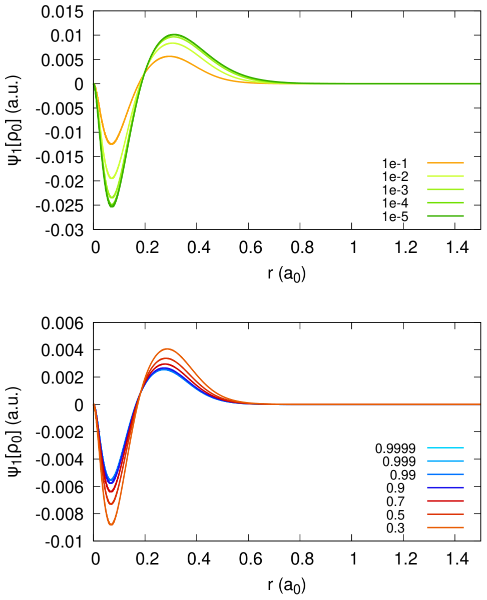

FIG. 35. The quantity $\psi_1[\rho_0](r;\alpha)$ in the limit $\alpha \to 0^+$ (top) and $\alpha \to 1^-$ (bottom) obtained for the Al^{10+} ion, within the LDA

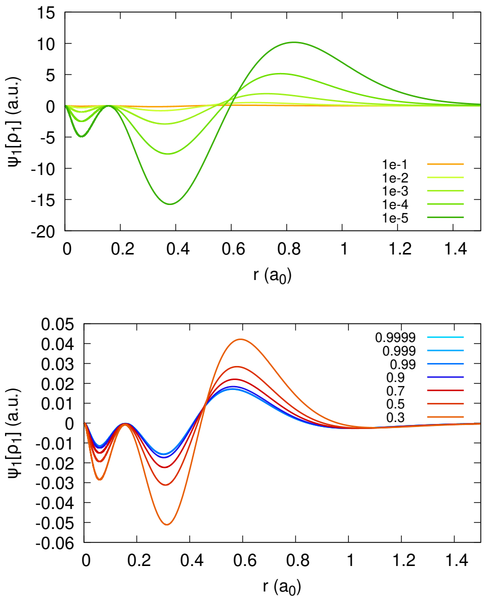

FIG. 36. The quantity $\psi_1[\rho_1](r;\alpha)$ in the limit $\alpha \to 0^+$ (top) and $\alpha \to 1^-$ (bottom) obtained for the Al^{10+} ion, within the LDA
